# Electrospun Liquid Crystal Elastomers as Stress-Free Thermo- and Photoresponsive Actuators

**DOI:** 10.1021/acsami.6c09275

**Published:** 2026-07-05

**Authors:** Niccolò Braidi, Michele Zanoni, Marco Turriani, Ruggero Rossi, Carla Triunfo, Daniele Martella, Stefano Masiero, Camilla Parmeggiani, Chiara Gualandi

**Affiliations:** † Department of Chemistry “Giacomo Ciamician”, 9296 University of Bologna, Via Piero Gobetti 83, Bologna 40129, Italy; ‡ European Laboratory for Non Linear Spectroscopy (LENS), via N. Carrara 1, Sesto Fiorentino 50019, Italy; § Department of Chemistry, “Ugo Schiff” University of Florence, via della Lastruccia 3−13, Sesto Fiorentino 50019, Italy; ∥ INSTM UdR of Bologna, University of Bologna, Via Selmi 2, Bologna 40126, Italy; ⊥ Interdepartmental Center for Industrial Research on Advanced Applications in Mechanical Engineering and Materials Technology, CIRI-MAM, University of Bologna, Viale Risorgimento, 2, Bologna 40136, Italy; # Health Sciences & Technologies (HST) CIRI, University of Bologna, Via Tolara di Sopra 41/E, Ozzano Emilia Bologna 40064, Italy

**Keywords:** thermal actuation, liquid crystal elastomers, electrospinning, photoresponsive polymers, smart materials

## Abstract

Actuators based on liquid crystalline elastomers (LCEs) can realize reversible and complex shape changes exploitable in soft robotics, actuators, surface haptics, photocontrolled microfluidic, and artificial muscles. A key prerequisite for the actuation of LCEs lies in predetermined alignment of mesogenic units. Here, we describe a single-step fabrication process for manufacturing LCE actuators using electrospinning coupled with simultaneous photo-cross-linking. Branched oligomers were designed and synthesized to address the trade-off between electrospinnability and cross-linking density. Moreover, optimizing the photoinitiator system and electrospinning setup allowed us to achieve long-range alignment of liquid crystalline domains. These fibrous materials display impressive stress-free thermal actuation without the need for external loads, reaching reversible deformations of over 30%. Additionally, we explored photoresponsive behavior by integrating photothermal dyes, enabling movement triggered by either UV or blue light. These results provide a scalable framework for developing smart materials suitable for applications in soft robotics and artificial muscles.

## Graphical abstract

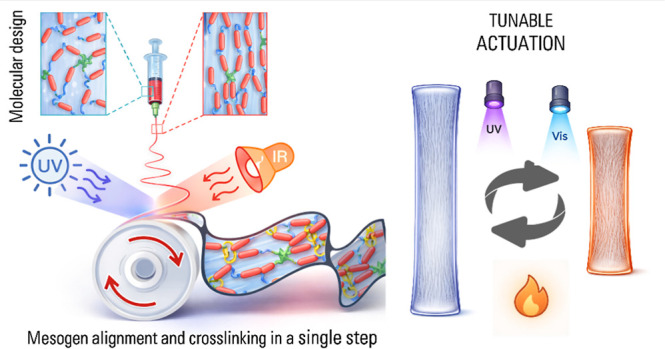

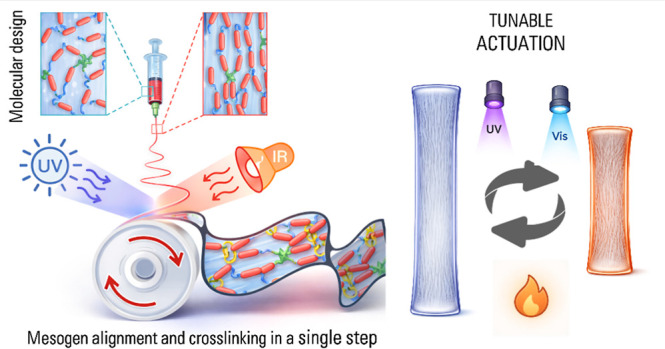

## 1 Introduction

Liquid crystal elastomers (LCEs) are cross-linked polymer networks where mesogens (units able to form liquid crystalline phases) are integrated into flexible backbones. Like other polymer-based responsive materials,^1,2^ LCEs are soft, compliant, and lightweight, making them attractive for applications in soft robotics, wearable systems, and medicine.^3–6^ Their ability to transduce external stimuli, such as heat, light,^7^ or electricity,^8^ into mechanical motion originates from the interplay between the entropic elasticity of the network and the orientational order of the mesogens.

When the mesogens are uniformly aligned along a common director, the polymer chains adopt elongated, thus anisotropic, conformations^4^ which can be fixed by binding the chains together, commonly via chemical cross-links. Upon heating (directly or with other stimuli) the mesogens gradually lose their orientational order until, in some cases, a nematic-to-isotropic phase transition occurs, the “anisotropic pull” on the chains vanishes, and the chains relax toward random-coil conformations. Because of the cross-links, this isotropization generates internal stress stored in the network with a consequent macroscopic contraction along the initial alignment direction. Upon cooling, the mesogens reorder, actively pulling the material back toward its original shape, enabling reversible shape recovery. Stress-free actuation, namely the ability to undergo a reversible shape change in the absence of any externally applied mechanical load, suspended weights, or other constraints that introduce stress, is governed by two key requirements in LCEs. First, a uniform long-range alignment of mesogens (ideally a monodomain) is required to translate the microscopic conformational changes at the nematic-to-isotropic phase transition into a coherent macroscopic shape change.^4,9^ Second, the cross-link density must be optimal: insufficient cross-linking results in weak internal stress and consequently in low recovery force, whereas excessive cross-linking freezes the liquid crystal order hindering thermal response.^3^

The formation of cross-linked and highly oriented liquid crystalline domains is challenging and has been achieved by adopting several fabrication strategies, including surface forces,^10,11^ electric or magnetic fields applied during curing,^12,13^ two-step spin-casting,^14^ microextrusion or direct ink writing,^15–17^ and microfluidics.^18–20^ However, most of these methods suffer from limitations such as the necessity of low-viscosity prepolymers, high material consumption, complex multistep procedures, or reliance on custom-built setups, thus hindering systematic studies, efficient process optimization, and scalability. To overcome these constraints, particularly the bottleneck imposed by custom-built instrumentation, additive manufacturing approaches, such as direct-ink writing, are increasingly being explored as scalable alternatives to conventional alignment cells.

In this context, fiber fabrication techniques may prove equally relevant, offering the possibility of producing aligned architectures over large areas without the constraints of custom-built systems.^18^ Moreover, LCE fibers have gained growing interest since, by taking advantage of the large specific surface areas, they display high response sensitivity, strong axial mechanical strength, and significant deformation flexibility, thus ensuring enhanced sensitivity and performance.^9^ Since the first report on LCE fibers obtained through polymer melt drawing,^21^ only a few studies have investigated the thermally induced shape change of oriented LCE fibers.^22–27^ Electrospinning, a high-throughput technology for producing fibers with high surface-to-volume ratios and tunable morphologies, has been recently proposed as an alternative fabrication technology to produce LCE fibrous actuators.^14,28–31^ Yet, few attempts to prepare LCE electrospun fibers have been reported. Reactive mesogens have been electrospun in the presence of a carrier polymer, either as a blend^28^ or in a core–sheath configuration,^29^ followed by photo-cross-linking in a subsequent step. Krause et al. developed a LC main-chain polymer incorporating benzophenone groups to enable UV-induced cross-linking, demonstrating that the nematic phase aligns along the fiber axis and that the fiber remains stable even above the clearing temperature.^14^ The electrospinning of reactive oligomers represents a more effective approach for actuation purposes. He et al. electrospun acrylate-terminated liquid-crystal oligomers under UV irradiation and obtained single thick fibers (fiber diameter >10 μm) capable of reversible thermal and photothermal actuation.^30^ Nevertheless, actuation was achieved only under the application of an external force, which was required to induce mesogen alignment along the fiber axis. Indeed, in their approach, the nematic domains were randomly distributed in the as-spun LCE fibers; therefore, actuation was possible only with the application of an external load applied to the fibers while stress-free actuation was not achieved. Similarly, Yang et al. could not obtain mesogen alignment during electrospinning of carbon nanotube/LCE yarns.^31^ To achieve actuation, after electrospinning, their fibers were mechanically stretched at a proper temperature to induce the formation of a monodomain that was fixed by photo-cross-linking.

Achieving stress-free actuation in as-spun fibers without the need of a post-treatment remains challenging, as it requires good mesogen alignment that must be fixed by a cross-linking reaction occurring immediately after fiber formation, or sufficiently fast during fiber formation, to prevent entropy-driven relaxation of the polymer chains.^32^ Consequently, fast cross-linking kinetics combined with slow relaxation dynamics are expected to favor mesogen alignment and enhance actuation performance. It is well-known that relaxation times decrease with increasing polymer molecular weight, which also promotes an entangled state in solution, enabling the production of continuous, bead-free fibers.^33^ Considering, as we noted earlier, that cross-link density is a critical parameter for fixing the mesogen orientation and achieving stress-free actuation, electrospinning of end-reactive prepolymers inherently presents a trade-off between spinnability and the attainable cross-link density in the resulting fibers.

In this work, we demonstrate the possibility to achieve stress-free actuation in as-spun fibers by adopting (i) a new molecular design based on electrospinnable branched liquid crystal oligomers and (ii) a fast photo-cross-linking reaction that occurs concomitantly with the electrospinning process. We first synthesized a series of branched, liquid crystal oligomers used to produce cross-linked electrospun fibers. We then proceeded with the optimization of the variables influencing photo-cross-linking, to achieve well-defined fibers with a high degree of mesogen order. Finally, the resulting nonwoven mats were actuated both under stress and in stress-free conditions to correlate their actuation properties with the network structure. Both thermal and photothermal actuations were tested, the latter enabled by the incorporation of photothermal agents.

## 2 Experimental Section

### 2.1 Materials

The following reagents and solvents were used as received without further purification: 2-methyl-1,4-phenylene bis­(4-((6-(acryloyloxy)­hexyl)­oxy)­benzoate) (RM257 – BLDpharm, 95%), 1,6-hexanedithiol (HexDT – BLDpharm, 98%), 1,8-diazabicycloundec-7-ene (DBU – BLDpharm, 98%), pentaerythritol tetrakis­(3-mercaptopropionate) (PeTT – Sigma-Aldrich, >95%), thioxanthen-9-one (TX – BLDpharm, 95%), *N*-ethyl-*N*-(2-hydroxyethyl)-4-(4-nitrophenylazo)­aniline (DR1 – Merck, 95%, Figure S1), deuterated chloroform (Sigma-Aldrich, 99.96 atom %D), dichloromethane (DCM – Sigma-Aldrich, ≥99.9%), chloroform (Sigma-Aldrich, ≥99.8%, amylenes as stabilizer). Additionally, 4-(4-nitrophenylazoyl)-phenol (HNAB, Figure S1) was synthesized as previously reported.^34,35^

### 2.2 Synthesis and Fabrication

#### 2.2.1 Synthesis of Linear Oligomers

RM257 (20.0 g, 33.9 mmol), HexDT (3.40 g, 22.6 mmol), DBU (337 mg, 2.22 mmol), and DCM (120 mL) were transferred to a 500 mL round-bottom flask equipped with an Allihn condenser. The reaction mixture was stirred in a thermostated oil bath (40 °C, 48 h). Afterward, the product was film-cast on a polytetrafluoroethylene (PTFE) sheet and then placed under vacuum until constant weight—the dry product was stored in a refrigerator. Different molecular weights of the acrylate-terminated telechelic oligomers (L) were achieved under the same reaction conditions by varying the RM257/HexDT molar ratio (Figure 1A, Table S1). Based on this ratio, we calculated the theoretical number-average molecular weight (*M*_n_^th^), under the assumption of complete conversion, using eq 1.

1 $$M_{n}^{th}=\frac{Z}{1-Z}\cdot \left({MM}_{RM257}+{MM}_{HexDT}\right)+{MM}_{RM257}$$

where: *Z* = $\frac{N_{HexDT}}{N_{RM257}}$ ; *N*_HexDT_ is the chemical quantity (mol) of HexDT; *N*_RM257_ is the chemical quantity (mol) of RM257, MM_HexDT_ is the molar mass of HexDT, and MM_RM257_ is the molar mass of RM257.

**Figure 1:**
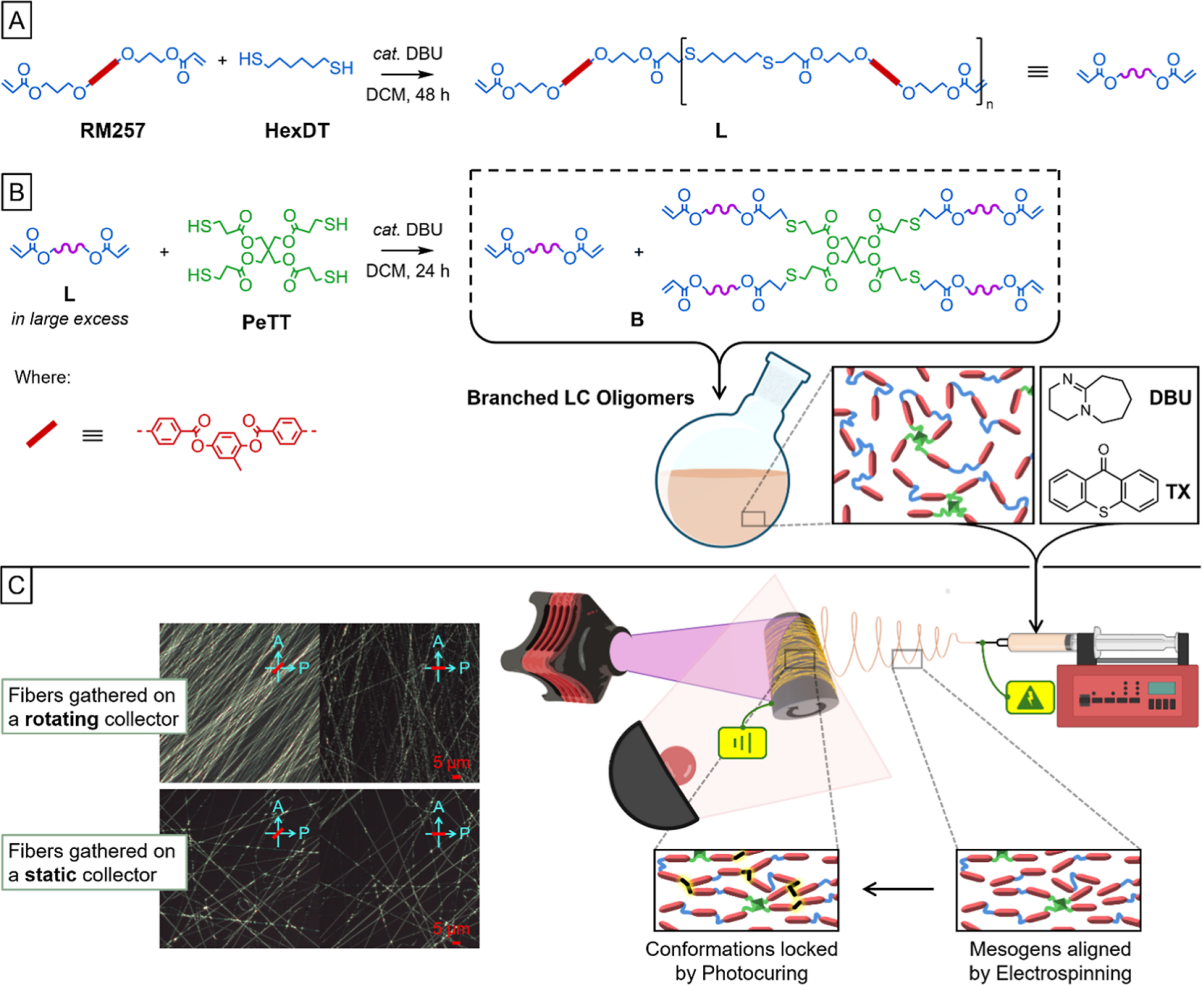
(A) Reaction scheme for the synthesis of linear diacrylate oligomers (L). (B) Reaction scheme for the synthesis of mixtures of branched oligomers and residual linear precursors (B). This simplified representation of B assumes, given the low percentages of PeTT employed, that there are no couplings between branched structures. B is subsequently mixed with the photoinitiator system (TX and DBU) for the preparation of electrospun formulations. (C) In the reported ES-PC process, the solution is electrospun onto a rotating cylindrical collector while being photo-cross-linked under UV light. As shown in the polarized optical microscopy (POM) images, the use of a rotating cylindrical collector allows the fibers to align, thus LC domains are preferentially oriented along the same axis.

#### 2.2.2 Synthesis of Mixtures of Branched Oligomers and Residual Linear Precursors

The corresponding reaction scheme is reported in Figure 1B. Small scale (∼500 mg). A linear oligomer (e.g., **L2** (*M*_n_^NMR^ = 2.26 kDa), Table S1, 500 mg, 0.221 mmol) was weighed into a vial and dissolved in DCM (1.47 mL). In a second vial, PeTT (21.64 mg, 0.0443 mmol) was dissolved in DCM (1.03 mL). Both solutions were cooled in a freezer at −20 °C before being slowly combined under vigorous stirring. Once homogenized, DBU (7.5 μL, 0.05 mmol) was added, and the reaction mixture was stirred in a thermostated oil bath (40 °C, 24 h). Afterward, the product (B) was film-cast in a PTFE Petri dish and then placed under vacuum until constant weight. The dry product was stored in a refrigerator. Different ratios of branched to residual linear oligomers were obtained under the same reaction conditions by varying the **L** to PeTT molar ratio (Table S2), ensuring the thiol-to-acrylate ratio (*R* = 2 *N*_PeTT_ *N*_**L**_^–1^) remained below 50% to prevent the formation of microgels. Batch scale (∼10 g). The same procedure was followed, but the oligomer solution was prepared in an appropriately sized flask and cooled in an ice-salt bath before adding the PeTT solution. Once homogenized, DBU was added, and an Allihn condenser was fitted on the flask. The reaction mixture was stirred in a thermostated oil bath (40 °C, 24 h). Afterward, the product was film-cast on a PTFE sheet and then placed under vacuum until constant weight. The dry product was stored in a refrigerator.

#### 2.2.3 Fabrication of Nonwoven Fabrics

All solutions for electrospinning were prepared using the following procedure. To a glass vial TX, DBU, oligomer, and chloroform were added alongside a stirring bar. The vial was wrapped in aluminum foil, and the oligomer solution was vigorously stirred at room temperature for 1 h before proceeding with the electrospinning process. As an example, the nonwoven fabric **M01** (Table S3) was prepared from 500 mg of oligomer **B7** (Table S2) that was dissolved in chloroform (1.43 mL) together with TX and DBU (40 mg each). Since the concentrations of oligomer, TX, and DBU were optimized in this study, the reader is referred to Tables S3 and S4 for the various quantities tested. Electrospinning was performed using a custom-built setup consisting of a SL 50 P10/CE/230 high-voltage power supply (Spellman, New York, USA), a KDS-200 syringe pump (KDScientific Inc., Massachusetts, USA), a glass syringe containing the oligomer/TX/DBU solution, and a stainless-steel needle (inner diameter of 0.51 mm) connected to the power supply set at 20 kV. The oligomer solution was dispensed at a rate of 1.0 mL h^–1^. For the grounded collector, either a static plate (6 × 6 cm^2^) or a rotating aluminum drum (⌀ = 3 cm, rotating at 5000 rpm) wrapped in parchment paper was used. In both cases, the needle was positioned 20 cm from the collector. For the static plate, the UV lamp (Thorlabs, 365 nm) was placed 50 cm away (intensity = 2 kW m^–2^), while for the rotating drum, the distance was set to 35 cm (intensity = 4 kW m^–2^). Additionally, in the case of the rotating drum, an IR lamp (PHILIPS 250 W) was pointed toward the collector ensuring a surface temperature of ∼50 °C, while the chamber was conditioned at 26 °C. The system is depicted in Figure S2. To perform the photoinduced actuation experiments (whether triggered by UV or visible light), the strips obtained from the mat were first washed with DCM and dried. These were then soaked in solutions containing the dye of interest (either HNAB or DR1) and dried again. By weighing the strips before and after dye loading, we determined the amount of dye incorporated into each sample.

### 2.3 Characterization Methods

#### 2.3.1 Nuclear Magnetic Resonance Spectroscopy (NMR)

^1^H NMR spectra were recorded on a Varian 400 MR NMR spectrometer (400 MHz) at 25 °C. The samples (∼25 mg) were dissolved in CDCl_3_ (∼0.5 mL). Chemical shifts (δ) are presented in parts per million and calibrated to the characteristic residual CHCl_3_ signal. The number-average molecular weight determined by ^1^H NMR (*M*_n_^NMR^) of the linear oligomers was calculated by applying eq 2, after imposing the value of the area attributed to the chain-end protons (*A*_D_ = 6).

2 $$M_{n}^{NMR}=\frac{A_{A}-4}{4}$$

where: *A*_A_ is the area of the signal attributed to the protons in the ortho position of benzoate moiety. Refer to Figures S6–S10 for the list of attributed signals and the ^1^H NMR spectra of **L1**-**L4**.

Given *M*_n,**L**_^NMR^ and the molar ratio of components employed (χ_**L**_) it was then possible to calculate the molecular weight of mixtures of oligomers reported in Table S4 (*M*_n, mix_) using eq 3.

3 $$M_{n,mix}=\sum_{L}\left(\chi_{L}\cdot M_{n,L}^{NMR}\right)$$

from this, we assume that after the reaction with PeTT, the system is composed of two molecular species, **B** and residual **L** (Figure 1B). The theoretical cross-linking density (ρ_X_^th^) is therefore defined as the maximum theoretical number of cross-linking units provided by both species (2 per residual **L** and 5 per **B** adduct), normalized by the total mass of the system.

$$\rho_{X}^{th}=\frac{2\cdot N_{L}+5\cdot N_{B}}{M_{n,mix}\cdot N_{L}+\left(4\cdot M_{n,mix}+{MM}_{PeTT}\right)\cdot N_{B}}$$

which, considering $R=2N_{PeTT}N_{{L_{0^{-1}}}^{-1}}$ and that *N*_**B**_ = *N*_PeTT_, results in eq 4.

4 $$\rho_{X}^{th}=\frac{4-3\cdot R}{2\cdot M_{n,mix}+{MM}_{PeTT}\cdot R}$$

#### 2.3.2 Gel Permeation Chromatography (GPC)

GPC was carried out using a KNAUER system equipped with a refractive index detector and a TSKgel SuperHM-M column (length = 150 mm and internal diameter = 6 mm). Tetrahydrofuran (THF) was used as an eluent with a 0.6 mL min^–1^ flow and sample concentrations of about 5 mg mL^–1^. The analyzed samples were solubilized in THF, stirred overnight, and filtered on a 0.450 μm PTFE filter. A molecular weight calibration curve was obtained with polystyrene standards in the molecular weight range 580–990,500 g mol^–1^.

#### 2.3.3 Thermogravimetric Analysis (TGA)

TGA measurements were conducted using a TA Instruments TGA Q500, from room temperature to 800 °C, under a nitrogen atmosphere. The dynamic temperature ramp applied (HiRes-TGA) was performed with sensitivity = 1, temperature ramp = 50 °C min^–1^, resolution = 4 °C.

#### 2.3.4 Differential Scanning Calorimetry (DSC)

DSC measurements were carried out using a TA Instruments Q2000 DSC, equipped with a refrigerated cooling system (RCS). The measurements were performed in a nitrogen atmosphere (50 mL min^–1^). To determine the glass transition (*T*_g_), crystal-to-nematic phase transition (*T*_CN_), and nematic-to-isotropic phase transition (*T*_NI_) temperatures, the samples were subjected to a first quench to −90 °C, followed by a first heating scan at 10 °C min^–1^ from −90 to 200 °C, a controlled cooling at 10 °C min^–1^ to −90 °C, and a second heating scan at 10 °C min^–1^ to 200 °C. *T*_g_ and *T*_NI_ were determined in the second heating scan; meanwhile, due to slow formation of the crystalline phase, *T*_CN_ was determined in the first heating scan.

#### 2.3.5 Scanning Electron Microscopy (SEM)

Samples were sputtered with gold and their morphology was recorded using a Leica Cambridge Stereoscan 360 at an accelerating voltage of 20 kV.

#### 2.3.6 Polarized Optical Microscopy (POM)

POM images of oligomer films, fibers collected on glass slides during spinning, and mats were obtained using a polarized optical microscope (Zeiss Axioscope) at various rotation angles relative to the polarization direction. Images at temperatures other than room temperature were acquired by placing the sample in a heating stage with an optical window (THMS600 with a T90 controller, Linkam).

#### 2.3.7 Attenuated Total Reflection Fourier Transform Infrared Spectroscopy (ATR FT-IR)

ATR FT-IR spectra of the mats were collected on a Bruker model Alpha spectrophotometer equipped with ATR, in the 4000–400 cm^–1^ range with a resolution of 3 cm^–1^.

#### 2.3.8 Two-Dimensional Wide-Angle X-ray Scattering (2D-WAXS)

The 2D-WAXS analysis of the samples was performed using a Bruker D8 Venture X-ray single-crystal diffractometer equipped with a PHOTON III-C14 area detector having a pixel size of 135 μm and a frame size of 728 × 1024 pixels. The samples were mounted with the sample length and width axes perpendicular to the beam (Ø = 200 μm) and parallel to the vertical and horizontal directions, respectively. Patterns were collected in still mode (θ = 0, ω = 0, χ = 0, φ = 0) at 300 K using a distance from the detector of 37.1 mm, 10 s exposure time and Cu–Kα radiation (λ = 1.54184 Å). The azimuthal profile at the value of 2θ identifying the Debye ring was obtained by integrating the intensity in the annular area near the 2θ using the software Diffrac.eva. The orientation factor, *f*, was then calculated from the corresponding azimuthal profiles *I*(φ) with Hermans’ eq (eq 5).

5 $$f=\frac{3\left\langle \cos^{2}\varphi \right\rangle -1}{2}$$

where

$$\left\langle \cos^{2}\varphi \right\rangle =\frac{\int_{0}^{\pi }I\left(\varphi \right)\cos^{2}\varphi \;\sin\varphi d\varphi }{\int_{0}^{\pi }I\left(\varphi \right)\sin\varphi d\varphi }$$

#### 2.3.9 Solubility Tests

Samples weighing 10–20 mg (*w*_start_) were added to a vial and immersed in 10 mL of dichloromethane overnight at room temperature. The retrieved sample was then dried until constant weight (*w*_end_). The obtained mats are named “post-swelling mats” and are free of the un-cross-linked soluble chains. Accounting for the amount of TX present in the sample (*w*_TX_), the insoluble fraction (Gel Content) was calculated using eq 6. Three replicates for each type of sample were performed and the mean values were reported.

6 $$Gel\;Content\left(\%\right)=\frac{w_{end}}{w_{start}-w_{TX}}\cdot 100$$

#### 2.3.10 Stress–Strain Tests

Tensile stress–strain measurements were performed by using a dynamic mechanical thermal analyzer (DMTA, TA Instruments Q800 series) equipped with tension-film clamps. All the analyses were performed on rectangular strips cut from electrospun mats (width = 5 mm; gauge length = 10 mm). The tests were conducted at 125 °C. Samples were heated to the target temperature and equilibrated for 5 min prior to testing. Stress–strain curves were then recorded using a force ramp of 0.005 N min^–1^.

#### 2.3.11 Actuation Tests

Actuation tests were performed using a DMA Q800 (TA Instruments) in tensile mode. The analysis was conducted on strips (5 mm wide) punched from the obtained mats. Prior to testing, the strips were thoroughly washed with DCM, deswelled in acetone, and air-dried. For purely thermal actuation tests, samples were mounted in the DMA, heated to 200 °C, and then cooled to 125 °C under a minimal normal force (0.001 N). The thermal history erasing step (200 °C for 5 min, then cooling to 125 °C) required approximately 15 min. Once thermal equilibrium at 125 °C was achieved, the sample length was recorded, and the desired prestretch was applied by increasing the normal force at a rate of 0.005 N min^–1^. Actuation experiments were performed both under stress-free conditions (prestretch = 0%) and under stress (prestretch >0%). Heating and cooling cycles were performed between 30 and 125 °C, each lasting about 20 min. For each prestretch value, five cycles were conducted. The actuation value (Actuation, eq 7) was averaged over the final three cycles, while the reversible recovery value (*R*_rev_, eq 8) was averaged over all five cycles. Refer to Figure S3 for the definition of ε_ON_, ε_OFF_, and ε_ON+1_.

7 $$Actuation\;\left(\%\right)=\varepsilon_{OFF}-\varepsilon_{ON}$$

8 $$R_{rev}\left(\%\right)=\frac{\varepsilon_{OFF}-\varepsilon_{ON+1}}{\varepsilon_{OFF}-\varepsilon_{ON}}\times 100$$

#### 2.3.12 Photoactuation under UV Light

For photoactuation tests, the DMA furnace was left open and a UV lamp (365 nm, 4 cm away from the sample, spot dimension: ∼1.7 mm, 3 kW m^–2^) was pointed at the sample (Figure S4). The lamp was turned on and off every 15 s, allowing us to similarly calculate Actuation and *R*_rev_. A thermal camera (Optris Xi 400) was used to measure the temperature of the specimen on both the illuminated side and the opposite side.

#### 2.3.13 Photoactuation under Visible Light

The photoactuation under visible light experiments were carried out using a custom-built setup consisting essentially of a force transducer (WPI KG4, 0–50 mN scale) and two LED lamps (Thorlabs M470L5) emitting blue light (470 nm) as shown in Figure S5. The samples (2.5 × 15 mm^2^) were vertically attached to the force transducer and kept under isometric conditions by a fixed metal bar. During the experiments, the samples were intermittently illuminated on both sides with a light intensity ranging from 10.8 to 15.9 mW mm^–2^. The LED control and force recording were managed by a LabVIEW program with a multichannel driver. The temperature of the samples during irradiation was recorded using a FLIR A500 streaming thermal imaging camera.

## 3 Results and Discussion

### 3.1 Optimization of the Reactive Electrospun Formulations

The aim was to develop a high-throughput process that relies on electrospinning coupled with concomitant photo-cross-linking (ES-PC) to produce LCEs capable of stress-free actuation. The process employs soluble mixtures of end-reactive branched liquid crystalline oligomers obtained via thia-Michael polyaddition of a mesogenic diacrylate (RM257), a dithiol chain extender (HexDT), and a tetrafunctional branching agent (PeTT) in the presence of DBU in a two-step synthesis (Figure 1A and B). These acrylate-terminated oligomers were combined with a Type II photoinitiating system of TX and DBU. TX acts as the near-UV/visible-absorbing triplet photosensitizer, while DBU is expected to act as the electron/hydrogen donor that generates the initiating α-aminoalkyl radicals, consistent with established TX/amine systems.^36–38^ Although Type II initiation is intrinsically bimolecular and therefore slower than Type I unimolecular cleavage, this system was selected in this work for two formulation-specific advantages. First, the amine donor mitigates oxygen inhibition, as peroxyl radicals abstract labile α-amino hydrogens to regenerate initiating radicals while consuming O_2_.^39,40^ Second, TX absorbs at longer wavelengths than previously employed Type I initiators, improving light penetration and limiting optical competition with the UV-absorbing RM257 mesogen.^41,42^

The resulting solution is thus electrospun onto a rotating cylindrical collector while the stretched polymers are simultaneously cross-linked under UV light (Figure 1C). Since stress-free actuation emerges within a specific range of cross-link densities (ρ_X_),^3^ optimization of the ES-PC process was required to overcome two conflicting properties of the oligomer solution: spinnability and cross-linking rate. For end-reactive linear polymers, both properties are inversely correlated to each other and strongly dependent on the oligomer molecular weight (MW). In other words, a higher MW improves spinnability since it decreases the critical entanglement concentration (*C*_e_)^43,44^ while it decreases the concentration of end-reactive functionalities and consequently the cross-linking rate.^45^ To solve this trade-off, we adopted the strategy of introducing branching points prior to electrospinning by using PeTT, with the specific aim of tuning ρ_X_ without compromising either spinnability or cross-linking rate.

We started our investigation by synthetizing linear diacrylate oligomers (**L**) with different molecular weights (Figure 1A, Table S1, Figures S11 and S12). At an arbitrary concentration ([oligomer]_0_ = 0.3 g mL^–1^ in chloroform), electrospinning of **L1** and **L2** (having *M*_n_^NMR^ = 1.00 and 2.26 kDa, respectively) yields droplets instead of continuous fibers; meanwhile beaded fibers are obtained from **L3** and **L4** (*M*_n_^NMR^ = 4.08 and 7.68 kDa, respectively). Due to the relatively low MW, these solutions fall in the semidilute unentangled regime ([oligomer]_0_ < *C*_e_). One possible strategy to reach the entangled regime is to increase [oligomer]_0_; however, as shown in Figure S13, even at [**L2**]_0_ = 0.7 g mL^–1^, the ES-PC process still yields beaded fibers. Therefore, by keeping the concentration at [oligomer]_0_ = 0.3 g mL^–1^, we added substoichiometric amounts of PeTT during synthesis (Figure 1B). Under these conditions, the thiol–acrylate reaction promotes chain extension and branching, thereby increasing the molecular weight and providing the viscoelastic properties required for electrospinning, while remaining below the gelation threshold to maintain solution processability throughout electrospinning. This shifts the solutions into the semidilute entangled regime as progressively higher amounts of PeTT transform the resulting morphology from droplets to bead-free fibers (Figure 2A). It can be noted that the lower the MW of the linear precursor, the higher the amount of PeTT required for fiber formation (the complete list of tested ratios and resulting morphologies is reported in Table S2). Another significant advantage of this strategy is that the final theoretical ρ_X_ in the resulting cross-linked material remains unchanged, regardless of the amount of PeTT, as ρ_X_ depends solely on the precursor’s MW. The amount of PeTT only controls the fraction of cross-linking points formed prior to the ES-PC process.

**Figure 2:**
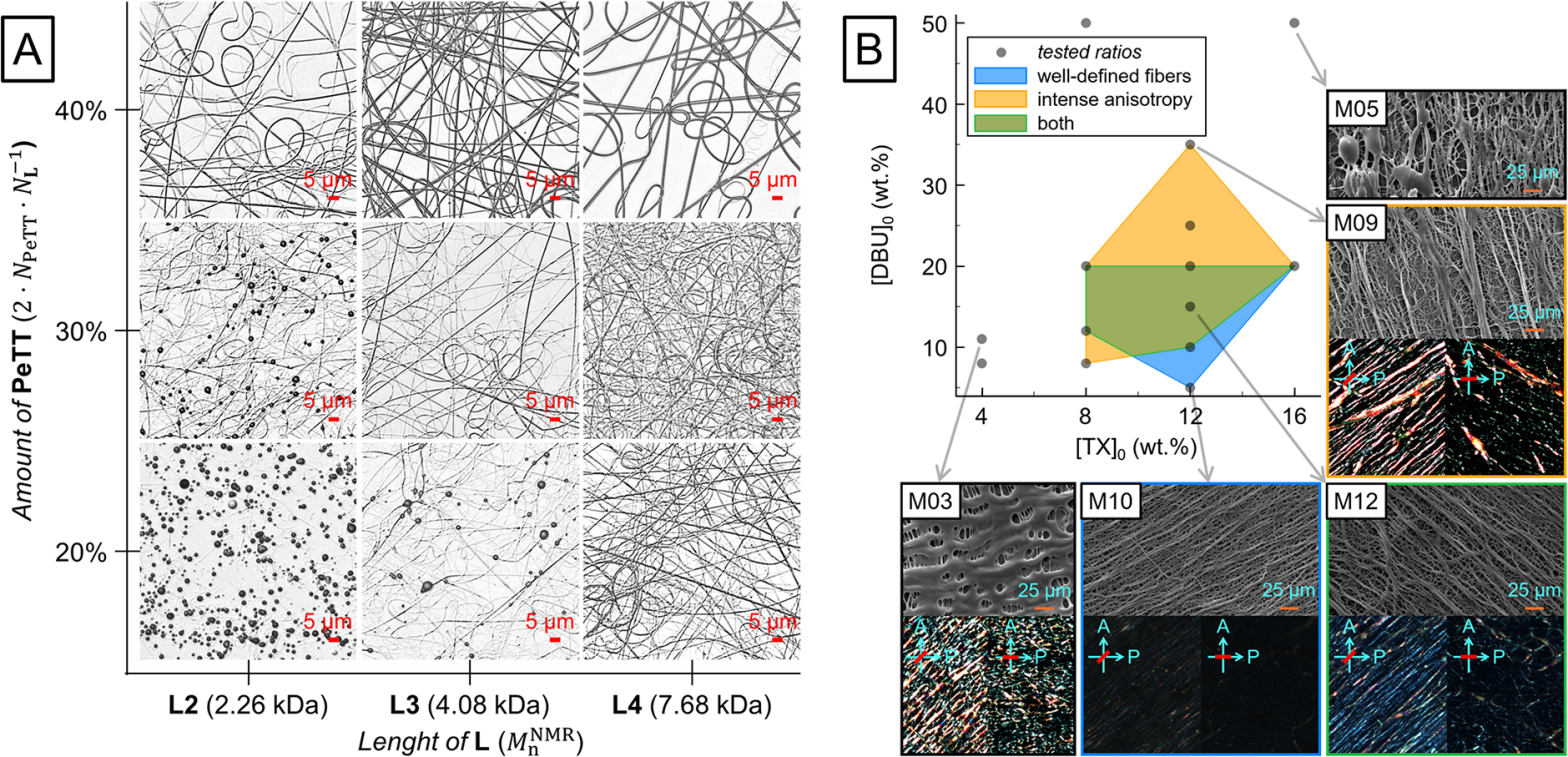
(A) Plot of pictures taken with an optical microscope which shows that increasing the amount of PeTT enables the formation of bead-free fibers, even at low MW of the strands between branching points (**L**). (B) Plot of [TX]_0_ vs [DBU]_0_ (relative weight with respect to oligomeric precursor), highlighted in green is the region yielding well-defined fibrous mats with strong birefringence. Representative SEM images (upper) and POM images (lower) of postswelling mats are shown; the complete series is provided in Figures S19–S23 and data in Table S3.

Another requirement for achieving stress-free actuation is that the LCE presents a well-defined macroscopic alignment;^18^ therefore, we subsequently targeted fast cross-linking kinetics. In the proposed ES-PC process, alignment of mesogens along the fiber direction is induced by the high shear forces experienced by the polymer chains during electrospinning.^14,32,46^ By using a rotating collector, a monodomain ensemble of liquid crystalline fibers can be obtained (Figure 1C). However, because the glass transition temperature (*T*_g_) of the oligomers employed is lower than room temperature (Figures S14–S18, Table S1), a slow cross-linking rate has a detrimental effect: newly deposited fibers have sufficient time to coalesce with the underlying layers, resulting in relaxation of the mesogenic alignment and consequently in a polydomain or poorly aligned material. Nevertheless, measuring the cross-linking rate in this system is challenging, considering that the oligomer concentration changes during the flight of the fiber due to solvent evaporation, along with variations in temperature and UV light intensity. Consequently, to evaluate the cross-linking rate, we relied on the qualitative observation of the resulting mats after treatment in dichloromethane (postswelling mats, free of the un-cross-linked soluble fraction), namely on the quality of fiber morphology (evaluated by SEM) and mesogen alignment (assessed by POM). We assume that both factors are strongly correlated with the cross-linking rate since high rates are expected to yield well-defined fibrous morphologies, rather than coalesced structures. Similarly, only sufficiently high cross-linking rates are expected to yield mats displaying strong anisotropic order under polarized light, as opposed to mats where polymer chains had sufficient time to relax their conformations. The optimization was conducted following a stepwise approach (Table S3). We systematically varied the [TX]_0_/[DBU]_0_ ratio, the MW of **L**, the oligomer concentration, and the use of an IR lamp to increase the temperature at the collector surface. The resulting SEM and POM images are reported in Figures S19–S23. The various experiments are also summarized in Figure 2B in the plot of [TX]_0_ vs. [DBU]_0_. More specifically, each mat obtained by spinning the formulations reported in Table S3 was classified according to the outcomes of SEM and POM analysis: the green color was assigned to mats with well-defined fibrous morphology exhibiting strong birefringence, the blue color corresponded to mats with good morphology but low birefringence, and the yellow color corresponded to film-like morphologies with high birefringence. A comparison between conditions **M01** and **M03** (IR lamp off) and **M02** and **M04** (IR lamp on) shows that IR-assisted heating significantly improves both fiber morphology and mesogen alignment (Figure S19). This result can be ascribed to the enhanced radical propagation kinetics and increased photopolymerization rate of acrylates at higher temperatures, driven by greater monomer mobility and decreased oxygen inhibition.^47,48^ Also, the photoinitiator couple plays a crucial role in determining both morphology and mesogen alignment. The optimal intervals of concentration are [TX]_0_ = 8–16 wt % and [DBU]_0_ = 12–20 wt % (Figure 2B). Within these [TX]_0_/[DBU]_0_ ratios, we then investigated the effect of the MW of **L** and found that good fiber morphology and mesogen alignment can be achieved by proportionally increasing the polymer concentration as the MW decreases (Figures S20–S23).

Given the optimized conditions, that allow us to produce well-defined fibrous mats with strong birefringence in a broad range of MW of the precursors, we took a further step in tuning the formulations by mixing the linear precursors (**L1**, **L2**, **L3**, and **L4**) at different molar ratios (Table S4, Figure S24) with the specific aim of targeting several cross-link densities (ρ_X_), the latter being a key parameter in controlling actuation performance. This enabled the preparation of fibrous mats with different theoretical cross-link densities (ρ_X_^th^ ranging from 0.39 to 2.28 mmol g^–1^, eq 4).

### 3.2 Thermal Actuation

Although in many LCE systems the DSC trace does not show a clear nematic-to-isotropic phase transition,^49–51^ in our case a transition peak around 95 °C is observed, which can be attributed to a nematic-to-isotropic phase transition (Figure S29). To further confirm this hypothesis and to better determine the actuation temperature range, a representative sample (**C12**, Table S4) was placed on a microscope slide coated with silicone oil and mounted on a heating stage to cycle the temperature from 30 to 130 °C (Video S1). By tracking the sample dimensions as a function of temperature, we determined the temperature range of actuation (Figure 3A). The first derivative of the fitted data in strain vs temperature shows that actuation occurs between 60 and 120 °C, with a maximum deformation rate at 95 °C.

**Figure 3:**
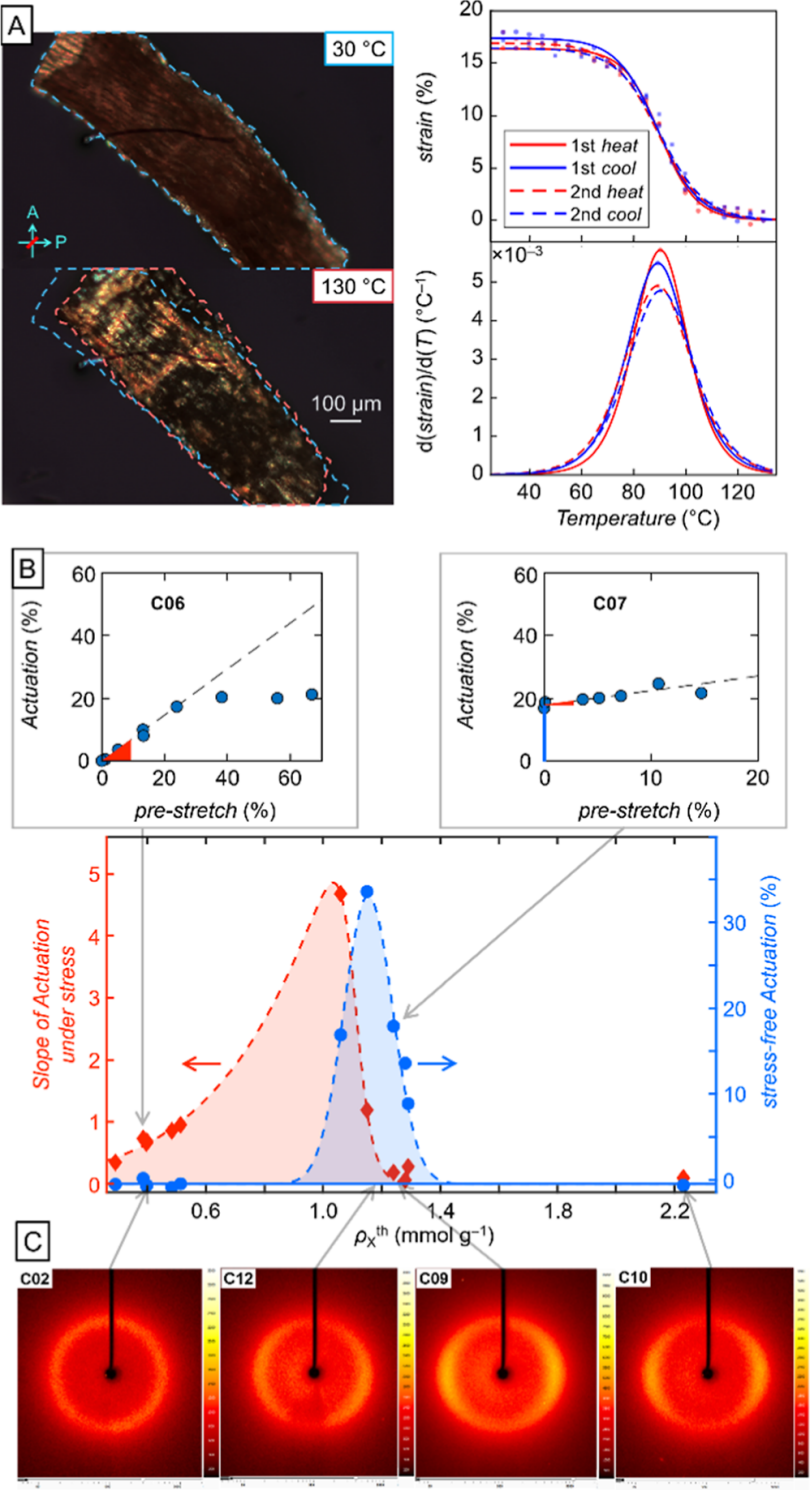
(A) On the left, POM images of a representative sample of C series collected at two temperatures (30 and 130 °C). The drawn traces (blue and red, respectively) highlight the stress-free actuation of the sample under temperature variation. Strain 0% is set at high temperature; during cooling, the sample elongated at strain >15%. Applying two cycles of heating and cooling (at 10 °C min^–1^) while acquiring images every 30 s allows the construction of the strain vs temperature plot shown on the right. The data are interpolated (lines), and the first derivative of the resulting function is used to identify the temperature range of actuation. (B) By applying different prestretches to the samples, Actuation vs prestretch plots are obtained (plots of **C06** and **C07** samples are reported as example). Linear interpolation of these data points allows the determination of the slope and intercept for each tested sample, with the latter corresponding to stress-free actuation, as a function of ρ_X_^th^ (red and blue, respectively) (Table S5). (C) 2D-WAXD analysis comparing the liquid crystalline orientation of representative samples of C series. The selected samples display increasing values of theoretical cross-linking density: ρ_X_^th^ of C02 = 0.399 mmol g^–1^; ρ_X_^th^ of C12 = 1.15 mmol g^–1^; ρ_X_^th^ of C09 = 1.29 mmol g^–1^; ρ_X_^th^ of C10 = 2.23 mmol g^–1^.

To perform accurate measurements of shape change, we employed a dynamic mechanical analyzer (DMA) applying thermal cycles from 30 to 125 °C at a heating/cooling rate of 10 °C min^–1^. Specifically, after erasing the thermal history, each sample was brought to 125 °C and subjected to a defined prestretch ranging from 0% (stress-free actuation) to 70% (see stress–strain tests at 125 °C of some selected samples in Figure S25). Afterward, it underwent five consecutive cooling and heating cycles. This procedure allowed the determination of the strain before and after thermal actuation (see an example of actuation in DMA, Figure S3), the Actuation, defined as the net strain induced by cooling (eq 7), and the reversibility of strain recovery (*R*_rev_), defined as the percentage of cooling-induced strain recovered upon heating (eq 8).^52^ The process was repeated at various prestretch values and, for each tested sample (Figure S24), Actuation and *R*_rev_ were plotted as a function of the prestretch (Figures S26–S29).

The data show that all samples exhibit positive Actuation under stress, namely when a prestretch >0% is applied, and that Actuation increases monotonically with increasing prestretch. As a general consideration, under load, the maximum attainable Actuation is strongly dependent on the applied prestretch, which is in turn limited by the maximum strain bearable by the material. Consistent with this, the highest Actuation observed in this study is 56% for sample **C12** (Table S5). Comparing this result with previously reported data on the Actuation of LC electrospun networks, some key differences emerge. In the work by He et al.^30^ electrospun microfibers achieved almost 60% of Actuation under stress. Yang et al.^31^ reported Actuation of approximately 70%. The latter is undoubtedly a noticeable result that however could be achieved only when electrospun yarns were uniaxially oriented by extremely high mechanical stretching (draw ratio of 9) before photo-cross-linking. In the present work, an Actuation of 56% is achieved without any post-treatment and applying a very modest prestretch (below 20%). While this result already represents a marked improvement for as-spun LCEs, a remarkable novelty emerges under stress-free conditions. Namely, several formulations display significant and fully reversible Actuation, sometimes above 30%, without the application of any mechanical loading (**C07–C09** and **C11–C12**, Table S5). An example of stress-free thermal actuation is reported in Video S1.

To better correlate the actuation performance to the LC network structure, from the Actuation vs prestretch plot, we extracted the slope of the initial linear region (Slope of Actuation Under Stress) which is then plotted against ρ_X_^th^ (red, Figure 3B). This bell-shaped curve shows that the slope increases progressively with increasing cross-link density, reaching a maximum at ρ_X_^th^ ∼1 mmol g^–1^. By further increasing ρ_X_^th^, the value rapidly drops to zero. Similarly, for each sample, we determined the stress-free Actuation (intercept of the initial linear region in the plot Actuation vs prestretch) and plotted it against ρ_X_^th^ (blue, Figure 3B). Here, stress-free Actuation was observed only within the narrower range ρ_X_^th^ = 0.90–1.40 mmol g^–1^, with a maximum of 33.6 ± 4.2% for sample **C12** (ρ_X_^th^ = 1.15 mmol g^–1^). The evolution of liquid-crystal orientation in representative samples of the C series was monitored by 2D-WAXD analysis. More specifically, diffractograms were collected on samples with increasing theoretical cross-linking density (Figure 3C). WAXS analyses show, for all samples, a wide diffraction peak located at 2θ = 19.6°. Sample C02, characterized by the lowest theoretical cross-linking density, exhibits a continuous and uniform Debye ring, with orientation factor f (eq 5) of 0.05, indicative of an essentially isotropic material. In contrast, the Debye rings of the other samples become anisotropic, demonstrating that a significant degree of liquid-crystal orientation is retained after the ES-PC process. Consistently, the value of the orientation factor, reported in Figure S30, together with the azimuthal plots, increases up to 0.51 for sample C12, indicating a partial but significant mesogen alignment; as the cross-linking density increases, the orientation factor also increases up to 0.68 and 0.61 for samples C09 and C10, respectively, suggesting a medium-to-high degree of uniaxial alignment of the mesogens. The results reported in Figure 3B,C indicate that, in the present system, significant mesogen alignment is achieved only above a critical theoretical cross-linking density. Furthermore, both stress-induced and stress-free actuation performances are strongly dependent on the cross-link density, in agreement with previous observations on bulk LCEs.^3^ More importantly, an optimal value of ρ_X_^th^ exists for maximizing the actuation response. At low ρ_X_^th^, cross-link are unable to lock in the aligned state, resulting in poor mesogen orientation and consequently limited actuation. Conversely, at high ρ_X_^th^, the increased cross-link density restricts chain mobility, thereby reducing the achievable actuation strain. Notably, the ρ_X_^th^ maxima of the Slope of Actuation Under Stress and the stress-free Actuation do not overlap. In addition, the two bell-shaped curves differ markedly in shape: the Slope of Actuation Under Stress is broader and asymmetric, while the stress-free Actuation is narrower and more symmetric. To the best of our knowledge, this type of analysis has not been reported previously; therefore, it should be validated across additional systems before proposing mechanistic explanations for these trends.

To demonstrate the scalability of the developed ES-PC process, stress-free actuation was also reproduced on a larger sample (7 × 1 cm^2^). By taking photographs at 30 and 130 °C, over 50 heating–cooling cycles, the sample strain, and consequently the stress-free Actuation and *R*_rev_, were determined (blue and green, Figure 4, respectively). As shown, the actuation reaches ∼36%, with barely any performance loss after 50 cycles. This behavior can only be ascribed to a high degree of mesogen alignment in the as-spun fibers and suggests that our developed ES-PC protocol (combining IR, UV, and an optimized [TX]_0_/[DBU]_0_ ratio) effectively freezes the mesogen configurations in a monodomain during fiber formation.

**Figure 4:**
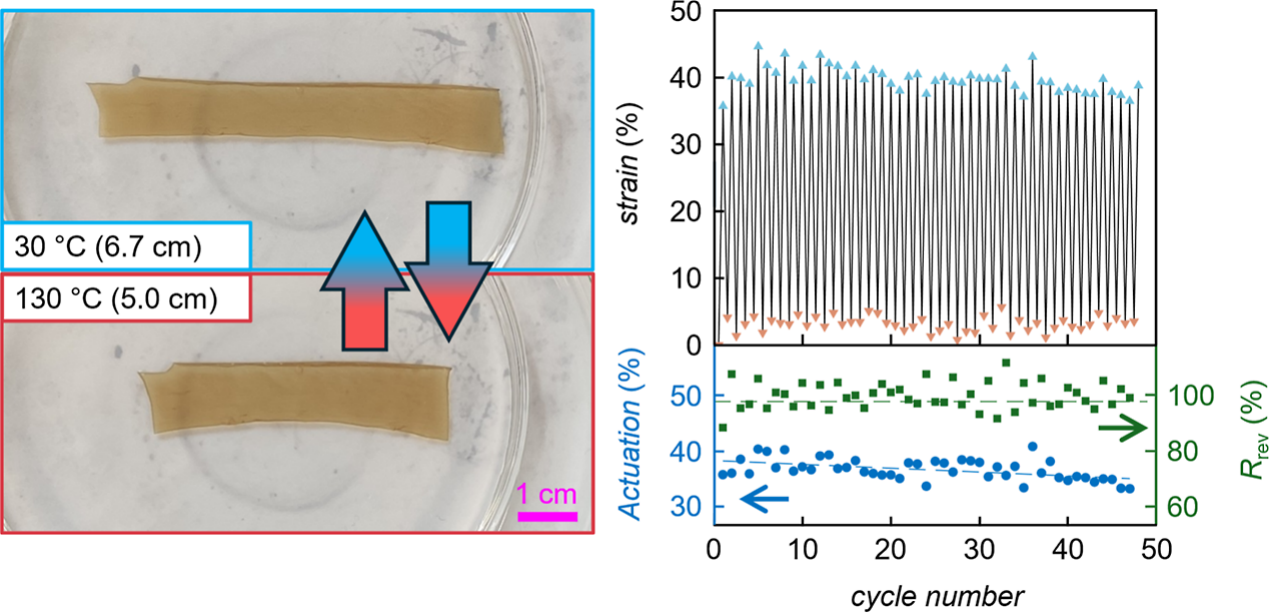
Left, photographs of a **C12** mat placed on a silicone-oil-coated Petri dish and actuated with a heating plate. Heating and cooling the sample, while acquiring images at the two extreme temperatures (30 and 130 °C), enables plotting of the strain variation over 50 cycles (right upper), from which Actuation and *R*_rev_ can be calculated and plotted (right lower).

### 3.3 Photoinduced Actuation Performance

We subsequently investigated the material response under light irradiation using two different setups: (i) a custom DMA add-on enabling sample irradiation under UV light while monitoring deformation (Figure S4), and (ii) a custom force-transducer setup to measure the force generated under visible blue-light irradiation in isometric conditions (Figure S5). Photosensitization of the samples was achieved by introducing suitable dyes in the fibers, namely 4-(4-nitrophenylazoyl)-phenol (HNAB) for irradiation at 365 nm^52^ and *N*-ethyl-*N*-(2-hydroxyethyl)-4-(4-nitrophenylazo)­aniline (DR1) for irradiation at 470 nm^53,54^ (Figure S1).

Because of the presence of UV-absorbing mesogens, the pristine mat is capable of converting UV radiation into heat, albeit the achieved temperature is not high enough for complete isotropization: at [HNAB] = 0 wt % the sample reaches an average temperature of 105 °C (determined with a thermal camera). The same sample loaded with azobenzene ([HNAB] = 6 wt %) reaches an average temperature of 117 °C, high enough to achieve actuation. By monitoring stress-free Actuation over 50 cycles of 15 s lamp-on followed by 15 s lamp-off, both samples exhibit a progressive decrease in actuation over cycling, which is appreciably more pronounced in the absence of azobenzene (Figure 5A). This may be due to polymer degradation induced by the combination of UV, heat, and atmospheric oxygen (Figure S31).

**Figure 5:**
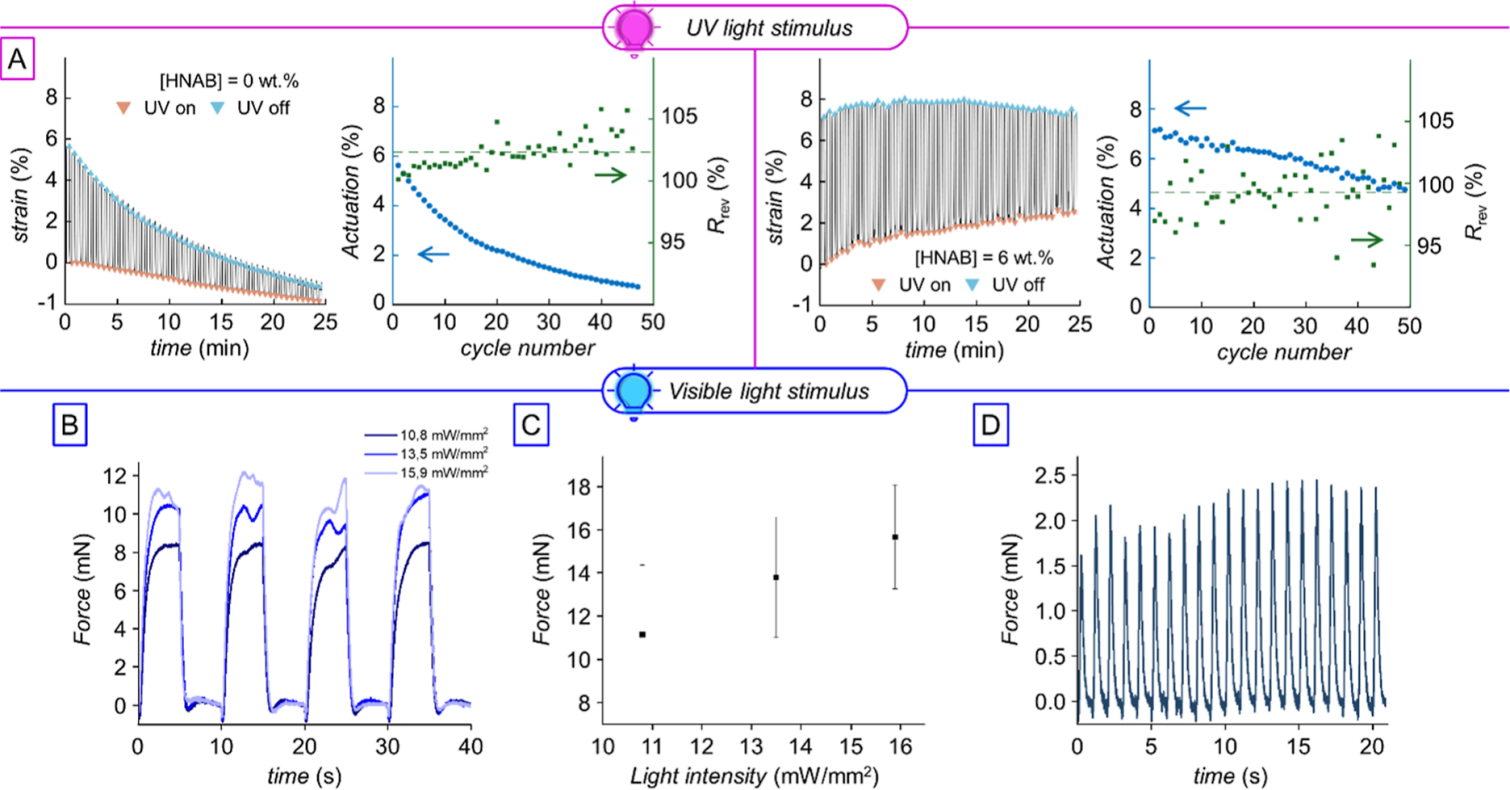
(A) Comparison of UV photothermal actuation of sample **C12** (Table S4) without and with the HNAB azobenzene dye (left and right plots, respectively): strain vs time upon alternating UV lamp on/off every 15 s; stress-free photothermal actuation and *R*_rev_ vs cycle number, derived from the corresponding time traces. (B) Force vs time traces recorded under blue-light actuation at increasing light intensities. (C) Average force values with error bars as a function of blue-light intensity. (D) Force traces under rapid light pulses (0.25 s, 15.9 mW mm^–2^).

In the second series of experiments, the photothermal additive was replaced to enable actuation at a lower-energy wavelength, thereby minimizing the risk of photodegradation due to UV exposure. We then tracked the force generated by a sample loaded with DR1 (0.6 wt %) produced under blue-light irradiation. The sample was clamped under isometric conditions, prestretched to 67% strain, and intermittently illuminated (5 s on, 0.1 Hz) while the generated force was recorded. After a brief initial relaxation, the samples began to develop force until reaching a maximum; upon removal of the light stimulus, complete relaxation occurred. Increasing light intensity produced a slight increase in generated force (Figure 5B,C) consistent with a high temperature reached by the sample during the irradiation cycle as reported in Figure S32. To demonstrate a fast actuation speed, as required for a possible application as artificial muscles,^55^ experiments were conducted also with higher-frequency light pulses at 15.9 mW mm^–2^ (0.25 s on, 1 Hz). Under these conditions, the samples did not reach a plateau force due to the short illumination time, instead producing a maximum force of 3.9 ± 1.6 mN (Figure 5D). Despite the higher frequency, the samples were able to follow the light stimulus without measurable hysteresis or degradation as already observed in similar conditions for other LCEs, and demonstrated the robustness and the potential of the approach here presented in all research fields requiring nonwovens with tunable actuation properties.^55^

## 4 Conclusions

In this work, we established a robust, single-step, high-throughput process to produce fibrous mats of liquid crystal elastomers capable of both under stress and stress-free actuation. Because the process relies on electrospinning coupled with concomitant photo-cross-linking, optimization had to address both spinnability and cross-linking rate, and (crucially) how to decouple the two. Our strategy relied on the introduction of branching points into the oligomers prior to electrospinning, using a tetrafunctional thiol. This increases the average molecular weight of the starting prepolymer before electrospinning. On the other hand, to maximize the cross-linking rate, we systematically varied the oligomer molecular weight and its relative concentration, photoinitiator ratio, and IR-assisted heating of the collector. This approach allowed us to identify an optimal processing window in which uniform-diameter fibers with long-range alignment of liquid crystalline domains could be consistently obtained. We then focused on thermal actuation tests. A series of oligomers with varying theoretical cross-link densities was synthesized, and the resulting mats were tested in DMA under repeated temperature cycles at different prestretches. Analysis revealed a narrow range of theoretical cross-link densities in which actuation (both under load and stress-free) reached its maximum, underscoring the central role of macromolecular architecture in dictating functional performance. In contrast, photothermal actuation experiments highlighted intrinsic material limitations under UV; using less energetic wavelengths (e.g., blue light), hysteresis or degradation were prevented even over 50 cycles. The ability to achieve reversible photoinduced deformation confirms the potential of these materials, while also defining the boundaries within which further improvements must be pursued. Taken together, our results demonstrate that electrospinning coupled with photo-cross-linking, and a proper macromolecular design, provides a powerful route to scalable fabrication of LCE mats with controllable actuation properties.

## Supplementary Material





## Notes

Experimental details, materials, methods, photographs of experimental setup; Characterization and properties of linear oligomers, branched oligomers, linear precursors, and mixtures of branched oligomers and residual linear precursors (PDF)
(MP4)

## |

N.B.: Department of Chemical and Geological Sciences, University of Modena and Reggio Emilia, via Campi 103, 41125 Modena, Ital.y

## References

[1] Zhang X., Wei J., Qin L., Yu Y. (2024). Liquid Crystal Polymer Actuators
with Complex and Multiple Actuations. J. Mater.
Chem. B.

[2] Qiu X., Zhang X. (2024). Self-healing Polymers
for Soft Actuators and Robots. J. Polym. Sci..

[3] Jiang Z.-C., Liu Q., Xiao Y.-Y., Zhao Y. (2024). Liquid Crystal Elastomers for Actuation:
A Perspective on Structure-Property-Function Relation. Prog. Polym. Sci..

[4] Ohm C., Brehmer M., Zentel R. (2010). Liquid Crystalline
Elastomers as
Actuators and Sensors. Adv. Mater..

[5] Martella D., Nocentini S., Parmeggiani C., Wiersma D. S. (2020). Photonic Artificial
Muscles: From Micro Robots to Tissue Engineering. Faraday Discuss..

[6] Martella D., Tusa I., Tubita A., Negri A., Turriani M., Rojas-Rodríguez M., De Luna M. S., Menconi A., Parmeggiani C., Rovida E. (2025). Liquid Crystalline Networks Hamper
the Malignancy of Cancer Cells. Adv. Healthc.
Mater..

[7] Luo J., Xu J., Bisoyi H. K., Xue Z., Qiu W., Tang J., Lu H., Tang Y., Li Q. (2026). High-Performance Flexible Pyroelectric
Energy Harvesting System Enabled by Light-Driven Thermomechanical
Coupling in Liquid Crystal Elastomer. Adv. Mater..

[8] Song R., Lv J. (2025). Electroactuated Liquid Crystal Polymers. Responsive
Mater..

[9] Zhang Z., Li J., Zhang R., Chen R., Zhang Y., Wang T., Yang K., Zhu J. (2024). Recent Advances in Responsive Liquid
Crystal Elastomer-contained Fibrous Composites. Responsive Mater..

[10] Stannarius R., Köhler R., Rössle M., Zentel R. (2004). Study of Smectic Elastomer
Films under Uniaxial Stress. Liq. Cryst..

[11] Küpfer J., Finkelmann H. (1991). Nematic Liquid
Single Crystal Elastomers. Makromol. Chem.,
Rapid Commun..

[12] Brehmer M., Zentel R., Wagenblast G., Siemensmeyer K. (1994). Ferroelectric
Liquid-crystalline Elastomers. Macromol. Chem.
Phys..

[13] Li M.-H., Keller P., Yang J., Albouy P.-A. (2004). An Artificial Muscle
with Lamellar Structure Based on a Nematic Triblock Copolymer. Adv. Mater..

[14] Krause S., Dersch R., Wendorff J. H., Finkelmann H. (2007). Photocrosslinkable
Liquid Crystal Main-Chain Polymers: Thin Films and Electrospinning. Macromol. Rapid Commun..

[15] Pozo M. D., Sol J. A. H. P., Van Uden S. H. P., Peeketi A. R., Lugger S. J. D., Annabattula R. K., Schenning A. P. H. J., Debije M. G. (2021). Patterned Actuators via Direct Ink Writing of Liquid
Crystals. ACS Appl. Mater. Interfaces.

[16] Lugger S. J. D., Verbroekken R. M. C., Mulder D. J., Schenning A. P. H. J. (2022). Direct
Ink Writing of Recyclable Supramolecular Soft Actuators. ACS Macro Lett..

[17] Simonetti G., Rossi R., Martella D., Credi C., Ferrantini C., Carpi F., Parmeggiani C. (2026). 4D Printing
of Fast-responsive Liquid
Crystalline Elastomers for Light-driven Actuators. Responsive Mater..

[18] Ohm C., Morys M., Forst F. R., Braun L., Eremin A., Serra C., Stannarius R., Zentel R. (2011). Preparation of Actuating
Fibres of Oriented Main-Chain Liquid Crystalline Elastomers by a Wetspinning
Process. Soft Matter.

[19] Stannarius R., Eremin A., Harth K., Morys M., DeMiglio A., Ohm C., Zentel R. (2012). Mechanical and Optical Properties of Continuously Spun
Fibres of a Main-Chain Smectic A Elastomer. Soft Matter.

[20] Shields A.
R., Spillmann C. M., Naciri J., Howell P. B., Thangawng A. L., Ligler F. S. (2012). Hydrodynamically Directed Multiscale Assembly of Shaped
Polymer Fibers. Soft Matter.

[21] Naciri J., Srinivasan A., Jeon H., Nikolov N., Keller P., Ratna B. R. (2003). Nematic Elastomer Fiber Actuator. Macromolecules.

[22] Lee J. E., Sun Y.-C., Naguib H. E. (2025). Multifunctional Soft Actuator Hybrids:
A Review. RSC Appl. Polym..

[23] Roach D. J., Yuan C., Kuang X., Li V. C.-F., Blake P., Romero M. L., Hammel I., Yu K., Qi H. J. (2019). Long Liquid
Crystal Elastomer Fibers with Large Reversible Actuation Strains for
Smart Textiles and Artificial Muscles. ACS Appl.
Mater. Interfaces.

[24] Sun J., Wang Y., Liao W., Yang Z. (2021). Ultrafast, High-Contractile
Electrothermal-Driven Liquid Crystal Elastomer Fibers towards Artificial
Muscles. Small.

[25] Liu J., Gao Y., Wang H., Poling-Skutvik R., Osuji C. O., Yang S. (2020). Shaping and
Locomotion of Soft Robots Using Filament Actuators Made from Liquid
Crystal Elastomer–Carbon Nanotube Composites. Adv. Intell. Syst..

[26] Nocentini S., Martella D., Wiersma D. S., Parmeggiani C. (2017). Beam Steering
by Liquid Crystal Elastomer Fibres. Soft Matter.

[27] Lin X., Saed M. O., Terentjev E. M. (2021). Continuous
Spinning Aligned Liquid
Crystal Elastomer Fibers with a 3D Printer Setup. Soft Matter.

[28] Yao J., Picot O. T., Hughes-Brittain N. F., Bastiaansen C. W. M., Peijs T. (2016). Electrospinning of Reactive Mesogens. Eur. Polym. J..

[29] Sharma A., Lagerwall J. (2018). Electrospun
Composite Liquid Crystal Elastomer Fibers. Materials.

[30] He Q., Wang Z., Wang Y., Wang Z., Li C., Annapooranan R., Zeng J., Chen R., Cai S. (2021). Electrospun
Liquid Crystal Elastomer Microfiber Actuator. Sci. Robot..

[31] Yang H., Wu D., Zheng S., Yu Y., Ren L., Li J., Ke H., Lv P., Wei Q. (2024). Fabrication and Photothermal Actuation
Performances of Electrospun Carbon Nanotube/Liquid Crystal Elastomer
Blend Yarn Actuators. ACS Appl. Mater. Interfaces.

[32] Richard-Lacroix M., Pellerin C. (2013). Molecular Orientation
in Electrospun Fibers: From Mats
to Single Fibers. Macromolecules.

[33] Shenoy S. L., Bates W. D., Frisch H. L., Wnek G. E. (2005). Role of Chain Entanglements
on Fiber Formation during Electrospinning of Polymer Solutions: Good
Solvent, Non-Specific Polymer–Polymer Interaction Limit. Polymer.

[34] Crespi S., Simeth N. A., König B. (2019). Heteroaryl
Azo Dyes as Molecular
Photoswitches. Nat. Rev. Chem..

[35] Telgerafchi A. E., Mehranpour M., Nazockdast H. (2018). Templated Assembly of Photoswitch
Azobenzene (4-(4-Nitrophenylazoyl)-Phenol) by Functionalization of
Multi-Walled Carbon Nanotube for Solar Energy Storage Applications. Chem. Phys. Lett..

[36] Yagci Y., Jockusch S., Turro N. J. (2010). Photoinitiated
Polymerization: Advances,
Challenges, and Opportunities. Macromolecules.

[37] Dadashi-Silab S., Doran S., Yagci Y. (2016). Photoinduced
Electron Transfer Reactions
for Macromolecular Syntheses. Chem. Rev..

[38] Nikitas N. F., Gkizis P. L., Kokotos C. G. (2021). Thioxanthone:
A Powerful Photocatalyst
for Organic Reactions. Org. Biomol. Chem..

[39] Ligon S. C., Husár B., Wutzel H., Holman R., Liska R. (2014). Strategies
to Reduce Oxygen Inhibition in Photoinduced Polymerization. Chem. Rev..

[40] Decker C., Jenkins A. D. (1985). Kinetic Approach of Oxygen Inhibition in Ultraviolet-
and Laser-Induced Polymerizations. Macromolecules.

[41] Ohzono T., Koyama E. (2022). Effects of Photo-Isomerizable
Side Groups on the Phase
and Mechanical Properties of Main-Chain Nematic Elastomers. Polym. Chem..

[42] Zhou J., Huang Y., Boromand A., Noori K., Purvis L., Oh C., Lu L., Ulissi Z. W., Gharakhanyan V., Zhang X. (2025). Genetic Algorithm-Accelerated Computational Discovery of Liquid Crystal
Polymers with Enhanced Optical Properties. RSC
Adv..

[43] McKee M. G., Wilkes G. L., Colby R. H., Long T. E. (2004). Correlations of
Solution Rheology with Electrospun Fiber Formation of Linear and Branched
Polyesters. Macromolecules.

[44] Andrady, A. L. Science and Technology of Polymer Nanofibers, 1 ed.; Wiley, 2008. 10.1002/9780470229842.

[45] Ehlers, F. ; Barth, J. ; Vana, P. Kinetics and Thermodynamics of Radical Polymerization. In Fundamentals of Controlled/Living Radical Polymerization; Tsarevsky, N. V. , Sumerlin, B. S. , Eds.; The Royal Society of Chemistry, 2013; pp 1–59. 10.1039/9781849737425-00001.

[46] Canejo J. P., Borges J. P., Godinho M. H., Brogueira P., Teixeira P. I. C., Terentjev E. M. (2008). Helical
Twisting of Electrospun Liquid
Crystalline Cellulose Micro- and Nanofibers. Adv. Mater..

[47] Scherzer T., Langguth H. (2005). Temperature Dependence of the Oxygen Solubility in
Acrylates and Its Effect on the Induction Period in UV Photopolymerization. Macromol. Chem. Phys..

[48] Setter R., Schmölzer S., Rudolph N., Moukhina E., Wudy K. (2023). Thermal Stability
and Curing Behavior of Acrylate Photopolymers for Additive Manufacturing. Polym. Eng. Sci..

[49] White T. J., Broer D. J. (2015). Programmable and Adaptive Mechanics with Liquid Crystal
Polymer Networks and Elastomers. Nat. Mater..

[50] Turriani M., Cosottini N., Fuochi N., Wiersma D. S., Martella D., Parmeggiani C. (2025). Exploiting
Photopolymerization to Modulate Liquid Crystalline
Network Actuation. Soft Matter.

[51] Donato S., Bini R., Simonetti G., Fuochi N., Salzano
De Luna M., Chatard C., Brient P.-L., Wiersma D. S., Martella D., Parmeggiani C. (2025). What Is the Right Chain Length? Liquid
Crystalline Network Tuning by Molecular Design. Macromolecules.

[52] Zanoni M., Cremonini A., Toselli M., Montalti M., Natali D., Focarete M. L., Masiero S., Gualandi C. (2024). Amplification
of Photothermally
Induced Reversible Actuation in Non-Woven Fabrics Compared to Bulk
Films. Sens. Actuators B Chem..

[53] Donato S., Martella D., Salzano De Luna M., Arecchi G., Querceto S., Ferrantini C., Sacconi L., Brient P., Chatard C., Graillot A., Wiersma D. S., Parmeggiani C. (2023). The Role of
Crosslinker Molecular Structure on Mechanical and Light-Actuation
Properties in Liquid Crystalline Networks. Macromol.
Rapid Commun..

[54] Martella D., Nocentini S., Micheletti F., Wiersma D. S., Parmeggiani C. (2019). Polarization-Dependent
Deformation in Light Responsive Polymers Doped by Dichroic Dyes. Soft Matter.

[55] Ferrantini, C. ; Pioner, J. M. ; Martella, D. ; Coppini, R. ; Piroddi, N. ; Paoli, P. ; Calamai, M. ; Pavone, F. S. ; Wiersma, D. S. ; Tesi, C. ; Cerbai, E. ; Poggesi, C. ; Sacconi, L. ; Parmeggiani, C. Development of Light-Responsive Liquid Crystalline Elastomers to Assist Cardiac Contraction. Circ. Res. 2019, 124(8). 10.1161/CIRCRESAHA.118.313889. 30732554

