# Supplementary material for: Electrospun Liquid Crystal Elastomers as Stress-Free Thermo- and Photoresponsive Actuators
Source: ACS Appl Mater Interfaces. 2026 Jul 5;18(28):39385–95. doi: 10.1021/acsami.6c09275 (PMC13397477; doi:10.1021/acsami.6c09275)
Supplement: Supplementary file 1 [file am6c09275_si_001.pdf]

SUPPORTING INFORMATION

Electrospun Liquid Crystal Elastomers as Stress-Free Thermo- and Photo-responsive Actuators

Niccolò Braidi,[$a] *Michele Zanoni,*[$a] *Marco Turriani,*[b] *Ruggero Rossi,*[b,c] *Carla Triunfo,*[a] *Daniele Martella,*[b,c] *Stefano Masiero,*[a] *Camilla Parmeggiani,*[b,c*] *Chiara Gualandi*[a,d,e,f,*]

a) Department of Chemistry "Giacomo Ciamician", University of Bologna, Via Piero Gobetti 83, 40129 Bologna, Italy
b) European Laboratory for Non Linear Spectroscopy (LENS), via N. Carrara 1, Sesto Fiorentino 50019, Italy
c) Department of Chemistry "Ugo Schiff" University of Florence, via della Lastruccia 3–13, Sesto Fiorentino 50019, Italy
d) INSTM UdR of Bologna, University of Bologna, Via Selmi 2, Bologna 40126, Italy
e) Interdepartmental Center for Industrial Research on Advanced Applications in Mechanical Engineering and Materials Technology, CIRI-MAM, University of Bologna, Viale Risorgimento, 2, Bologna 40136, Italy
f) Health Sciences & Technologies (HST) CIRI, University of Bologna, Via Tolara di Sopra 41/E, Ozzano Emilia Bologna 40064, Italy

[$] N.B. and M.Z. contributed equally to this paper

Corresponding author: Prof. Chiara Gualandi, email: c.gualandi@unibo.it; Prof. Camilla Parmeggiani, email: camilla.parmeggiani@lens.unifi.it.

Table of Contents

1. Experimental Section.....2

2. Characterization and properties of linear oligomers (L) 4

3. Characterization and properties of branched oligomers and residual linear precursors (B and M) 13

4. Characterization and properties of mixtures of branched oligomers and residual linear precursors (C) 20

5. Supporting Video.....28

1. Experimental Section

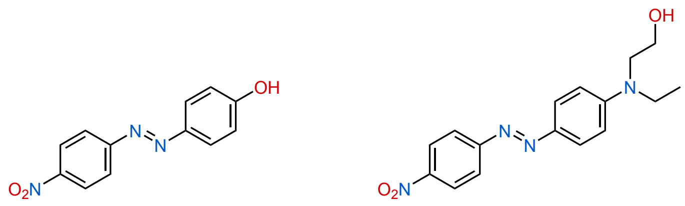

HNAB DR1

Figure S1. Chemical structure of the dyes employed in this work.

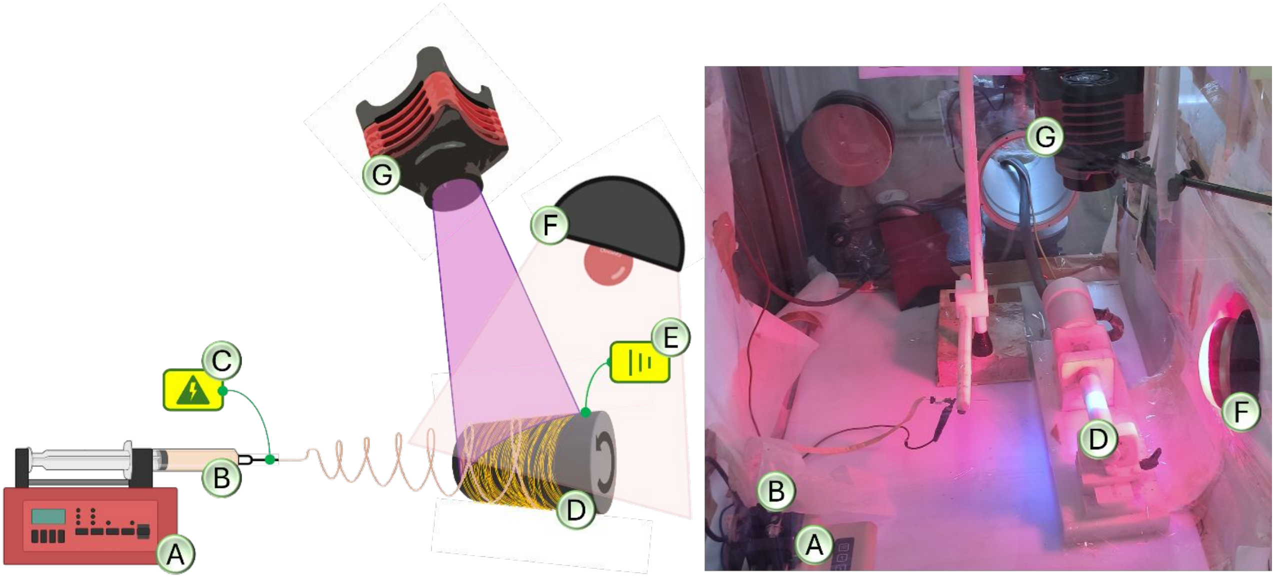

Figure S2. Scheme of the ES-PC system next to a picture of the actual system. **A**) syringe pump, **B**) a glass syringe containing the solution of oligomer, TX, and DBU in chloroform, **C**) high-voltage power supply connected to the syringe needle, **D**) rotating aluminum cylinder wrapped in parchment paper, used as the collector, **E**) connection between the collector and the ground, **F**) IR lamp (PHILIPS 250W), **G**) UV lamp (Thorlabs, 365 nm).

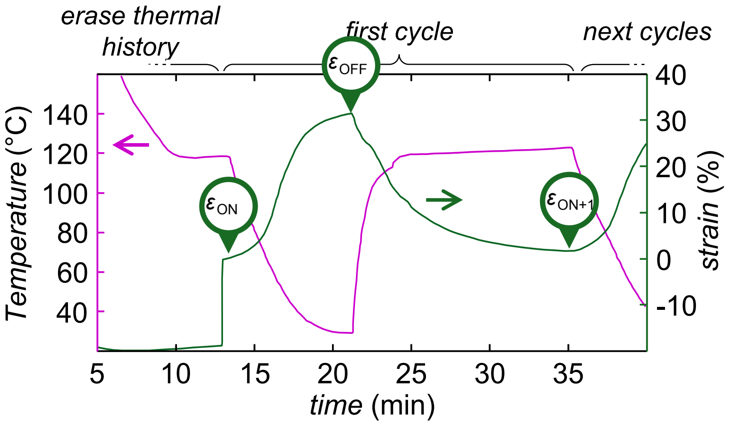

Figure S3. Representative plot of *Temperature* and *strain* vs. *time* obtained by an experiment in DMA, from which *Actuation* and R_{rev} can be determined using **Equation 7** and **8**, respectively, after determining ε_{ON}, ε_{OFF}, and ε_{ON+1}.

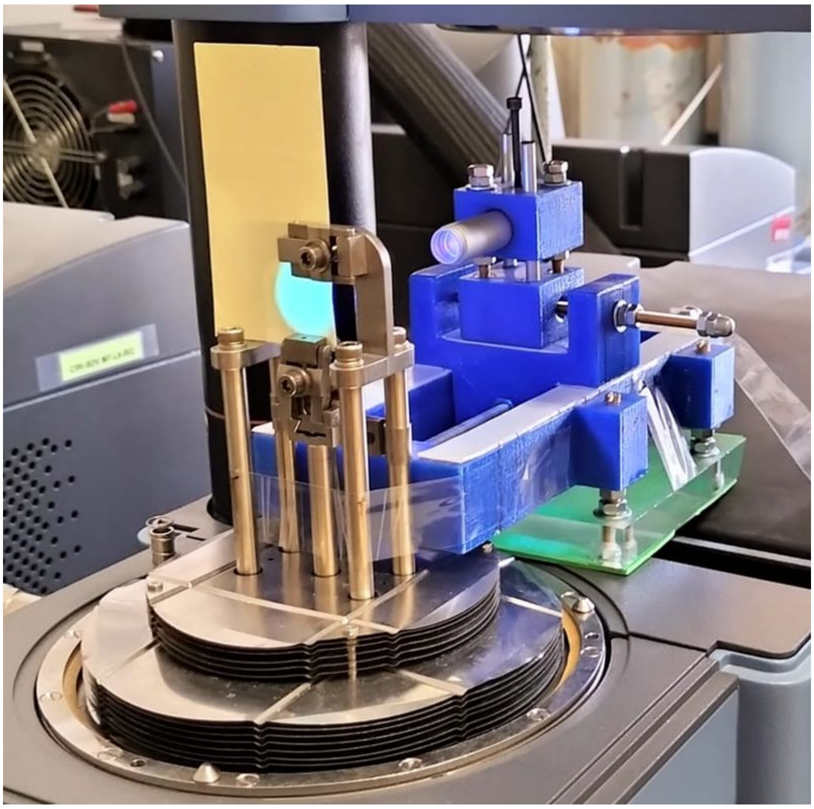

Figure S4. Photograph of the set-up employed to test photo-actuation performance under UV light.

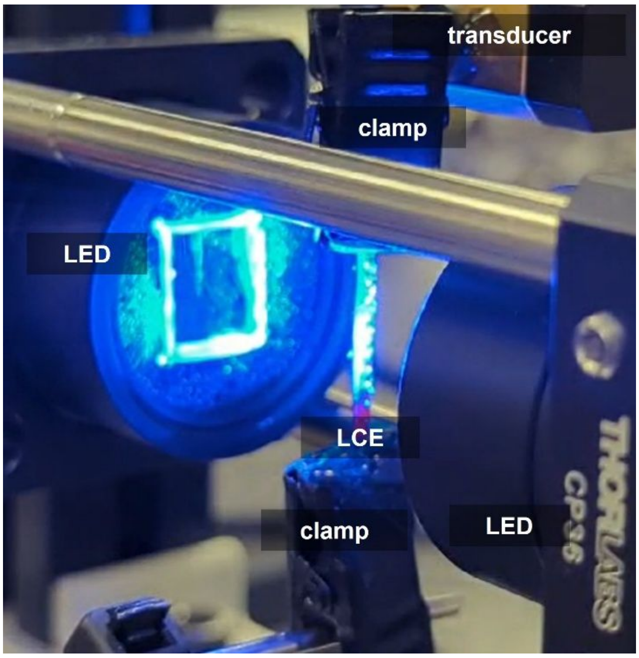

Figure S5. Scheme of the set-up employed to test photo-actuation performance under Visible light.

2. Characterization and properties of linear oligomers (L)

Table S1. Molecular weights (M_n) and thermal properties of linear diacrylate oligomers (**L**).[a]

entry	N_{RM257}/ N_{HexDT}	M_n^{th} [b] (kDa)	M_n^{NMR} [c] (kDa)	M_n^{GPC} [d] (kDa)	$Đ$ [d]	T_g [e] (°C)	T_{CN} [f] (°C)	T_{NI} [e] (°C)	T_d^{onset} [g] (°C)
**L1**	2.14	1.24	1.00	1.09	1.87	–11.8	44	112	300
**L2**	1.50	2.07	2.26	1.84	2.15	–7.6	69	106	314
**L3**	1.22	3.90	4.08	5.18	3.35	–5.1	77	104	306
**L4**	1.11	7.61	7.68	5.54	2.57	–0.7	84	105	308

a) Common conditions: DBU = 10 mol% with respect to HexDT, V_{DCM} = 120 mL, T = 40 °C, time = 48 h.
b) Calculated with **Equation 1**.
c) Calculated with **Equation 2**.
d) Determined by GPC.
e) Determined by DSC from the second heating scan (10 °C·min^{-1}).
f) Determined by DSC from the first heating scan (10 °C·min^{-1}).
g) Onset of degradation as determined by HiRes-TGA, under nitrogen.

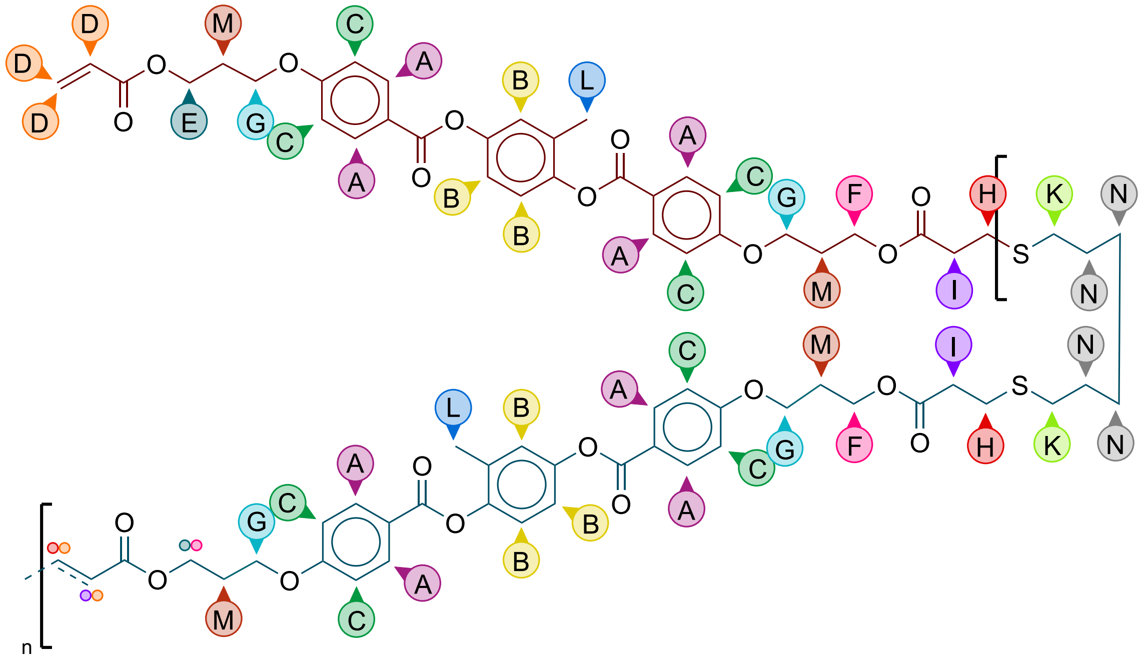

Figure S6. The structure of the linear oligomers obtained from the reaction between RM257 and HexDT. In ^{1}H-NMR, the following chemical-shifts have been attributed to: **A** = 8.15 ppm, **B** = 7.16 ppm, **C** = 6.98 ppm, **D** = 5.86–6.40 ppm, **E** = 4.38 ppm, **F** = 4.32 ppm, **G** = 4.14 ppm, **H** = 2.77 ppm, **I** = 2.62 ppm, **K** = 2.51 ppm, **L** = 2.24 ppm, **M** = 2.17 ppm, **N** = 1.38-1.78 ppm on the basis of 2D NMR correlations.

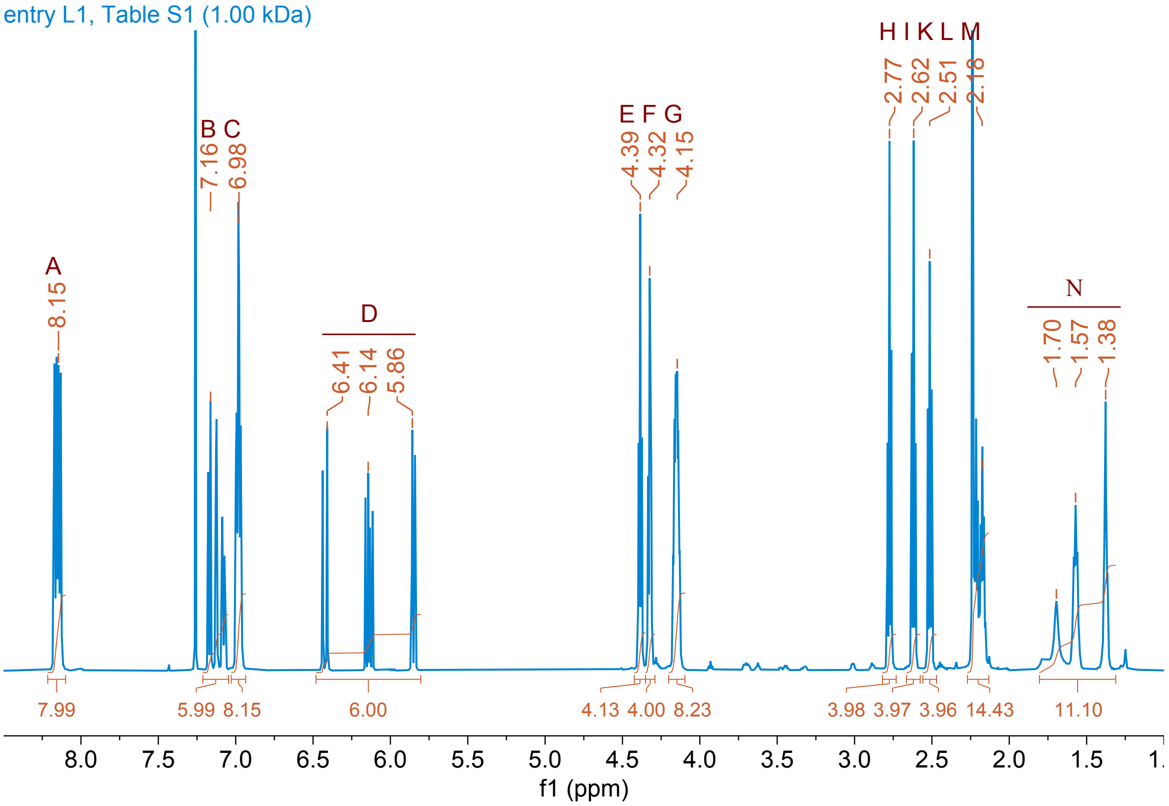

Figure S7. ^{1}H-NMR of the linear oligomer reported in **entry L1**, **Table S1** (M_n^{NMR} = 1.00 kDa).

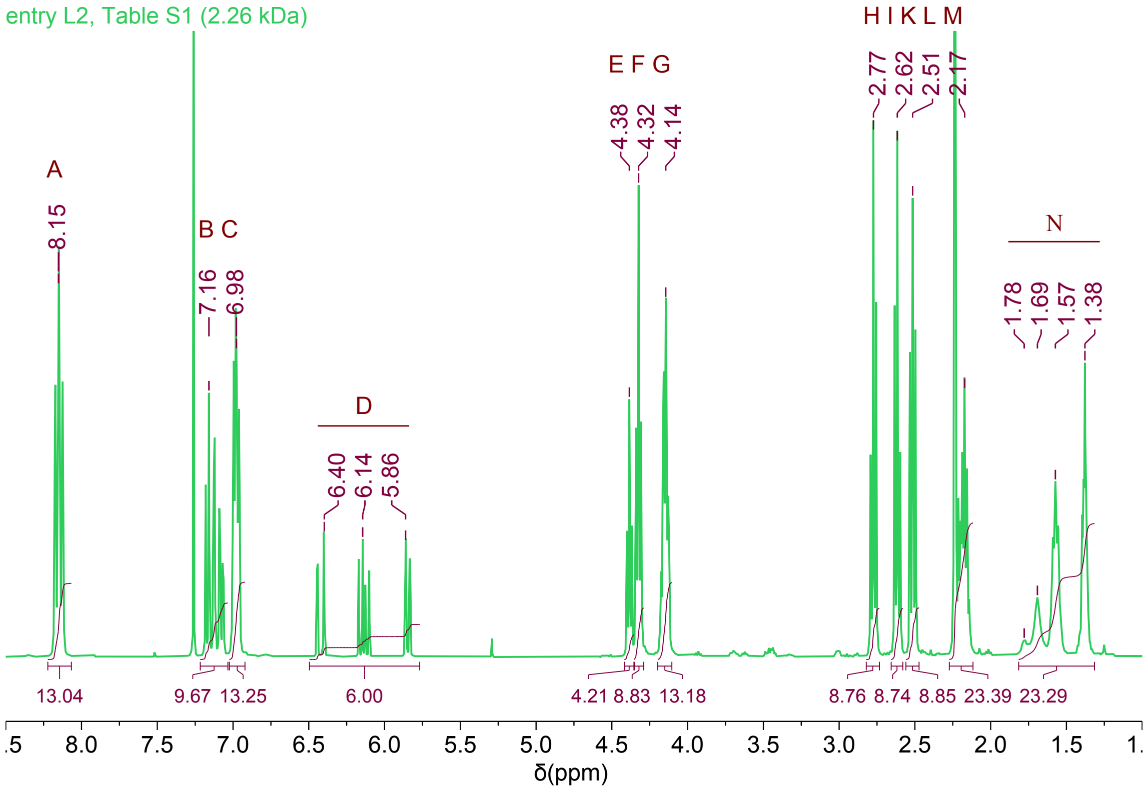

Figure S8. ^{1}H-NMR of the linear oligomer reported in **entry L2**, **Table S1** (M_n^{NMR} = 2.26 kDa).

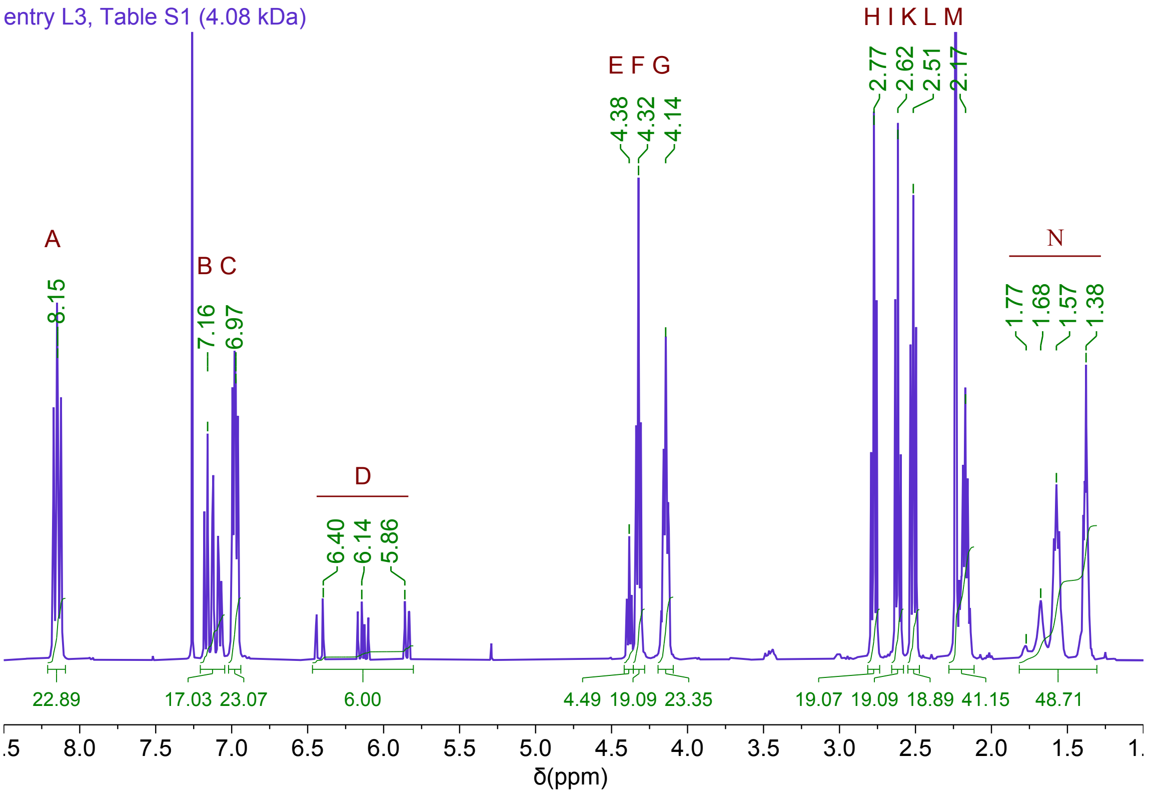

Figure S9. ^{1}H-NMR of the linear oligomer reported in **entry L3**, **Table S1** (M_n^{NMR} = 4.08 kDa).

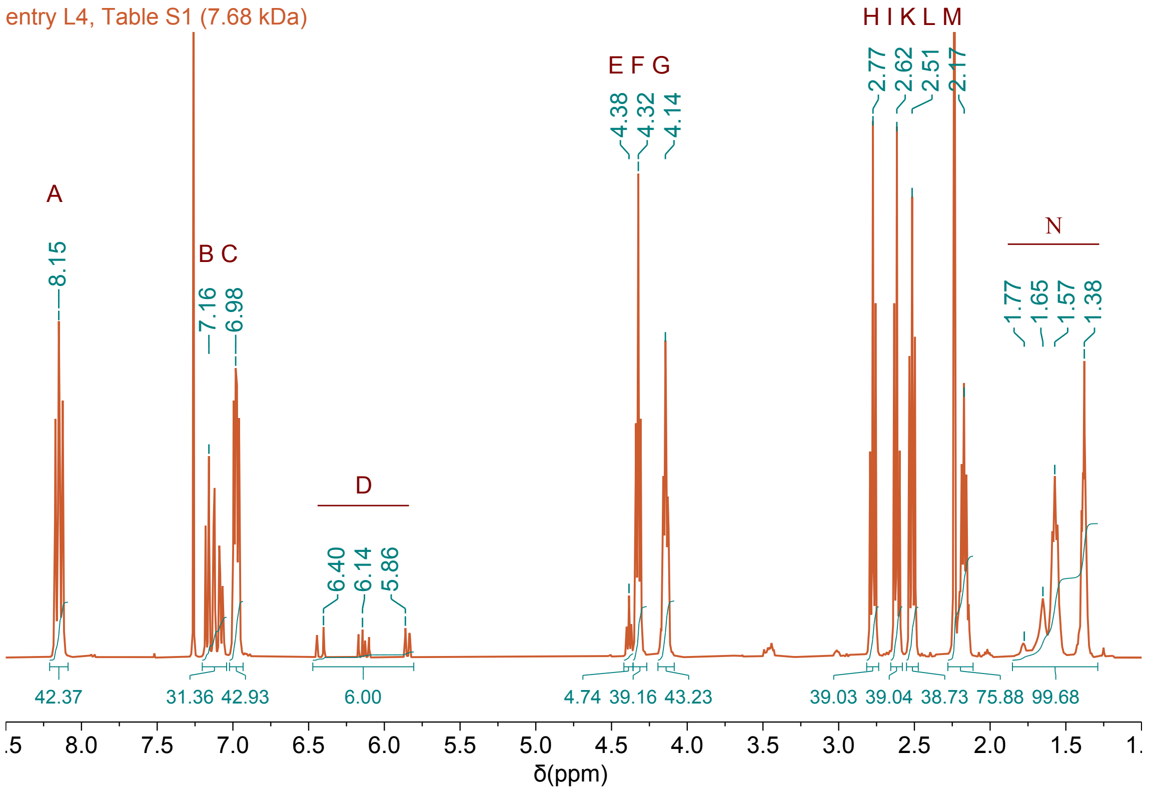

Figure S10. ^{1}H-NMR of the linear oligomer reported in **entry L4**, **Table S1** (M_n^{NMR} = 7.68 kDa).

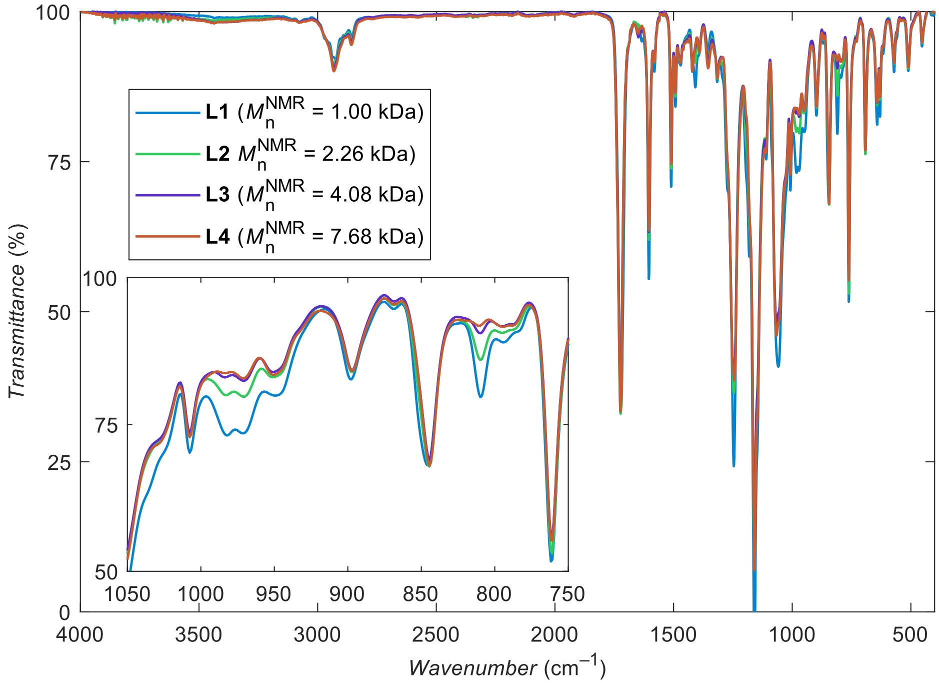

Figure S11. FTIR spectra of the linear oligomers reported in **Table S1**. The inset highlights the wavenumbers that show the highest change in transmittance.

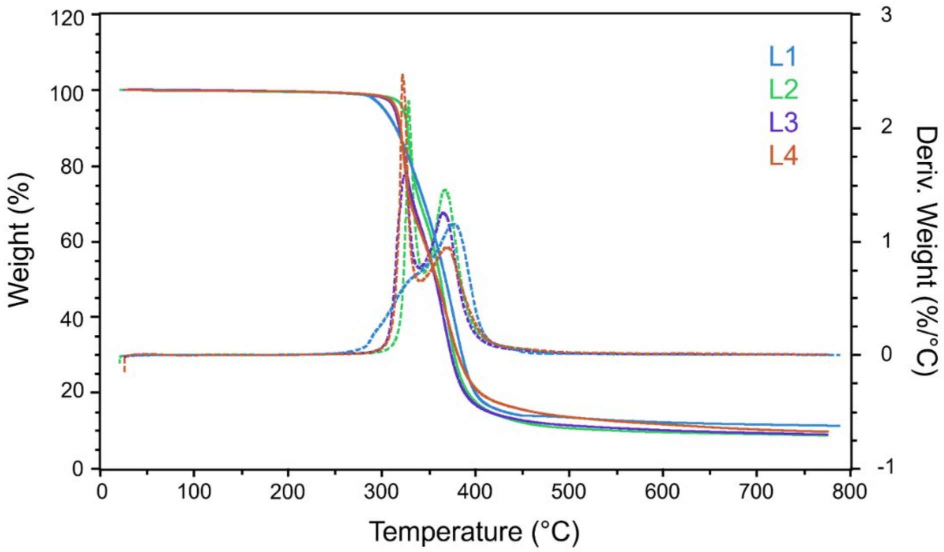

Figure S12. HiRes-TGA curves of the linear oligomers reported in **Table S1**. Percent weight loss against temperature (*continuous line*) and its derivative (*dashed line*).

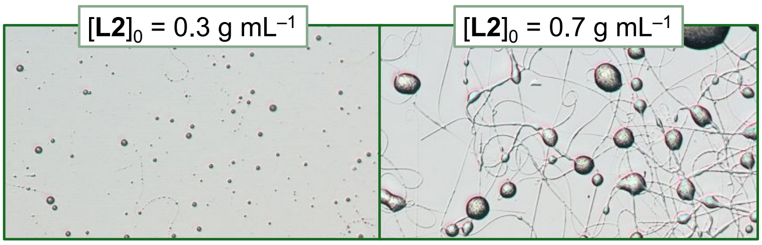

Figure S13. Optical microscopy of oligomer **L2** (**Table S1**) electrospun at two concentrations: 0.3 and 0.7 g mL^{-1} in chloroform.

Analysis of the thermal properties of **L1**–**L4** reveals that increasing oligomer M_n^{NMR} results in a progressive increase of the glass-transition temperature (T_g), from −11.8 to −0.7 °C. T_g was determined on the second heating scan, as we observed that from the metastable nematic phase these samples tend to reorganize into a crystalline phase; despite cooling at 10 °C min^{-1}, this crystalline phase does not quickly reform (**Figure S14**). Interestingly, the single melting endotherm observed in **L1** gradually evolves into two peaks of comparable enthalpy, separated by ~20 °C in **L2** and **L3**, while the higher-melting peak becomes dominant in **L4**. Conversely, the isotropization temperature appears unaffected by oligomer length, which causes a narrowing of the liquid-crystalline window with increasing M_n^{NMR}. These trends are corroborated by polarized optical microscopy (POM) images overlaid with DSC traces (**Figure S15–S18**), where a reduction in the size of the nematic domains is observed, likely due to reduced chain mobility associated with increasing viscosity in higher M_n^{NMR} liquid-crystalline oligomers.

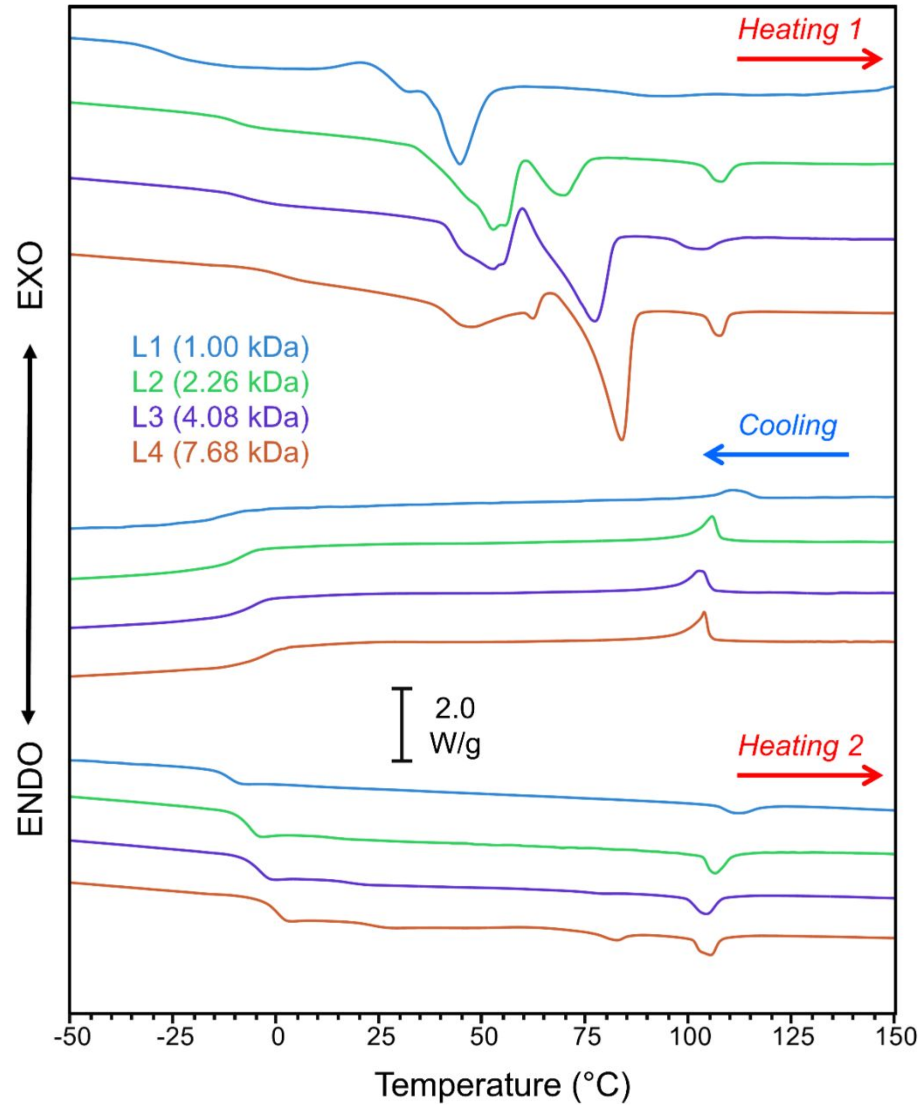

Figure S14. DSC curves of the linear oligomers reported in **Table S1**. From the top, first heating scan (10 °C·min^{-1}, from –90 °C to +200 °C), controlled cooling (10 °C·min^{-1}, from +200 °C to –90 °C), second heating scan (10 °C·min^{-1}, from –90 °C to +200 °C).

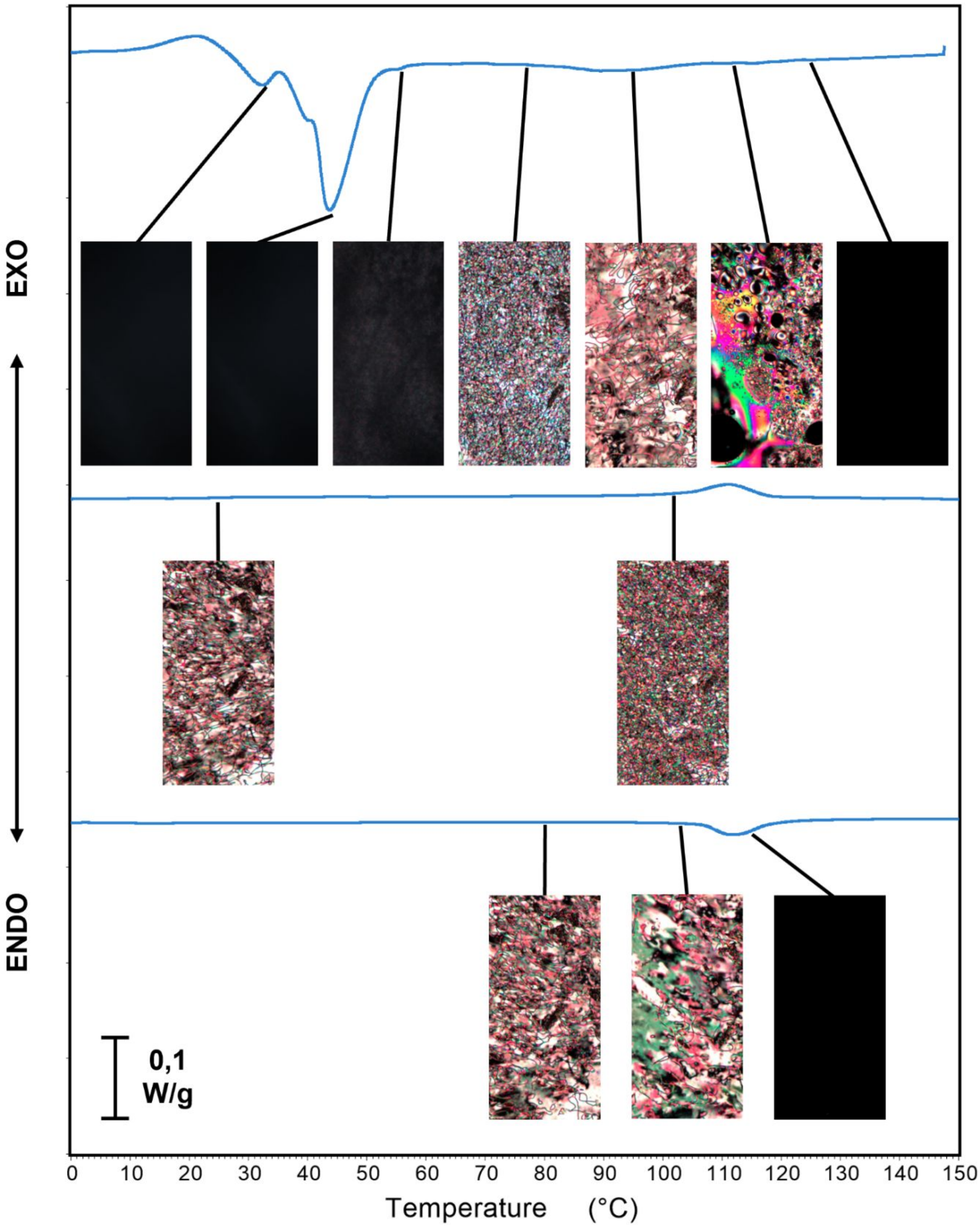

Figure S15. DSC curves and POM photographs of **L1** film, **Table S1** (M_n^{NMR} = 1.00 kDa). From the top, first heating scan (10 °C·min^{-1}, from –90 °C to +200 °C), controlled cooling (10 °C·min^{-1}, from +200 °C to –90 °C), second heating scan (10 °C·min^{-1}, from –90 °C to +200 °C). The temperature at which the POM images were collected was chosen based on the observed transition temperatures. For the first heating scan: T_{CN} = 44 °C, T_{NI} = 112 °C; for the cooling scan: T_{IN} = 110 °C, T_{NC} *undetected*; and for the second heating scan: T_{CN} *undetected*, T_{NI} = 112 °C.

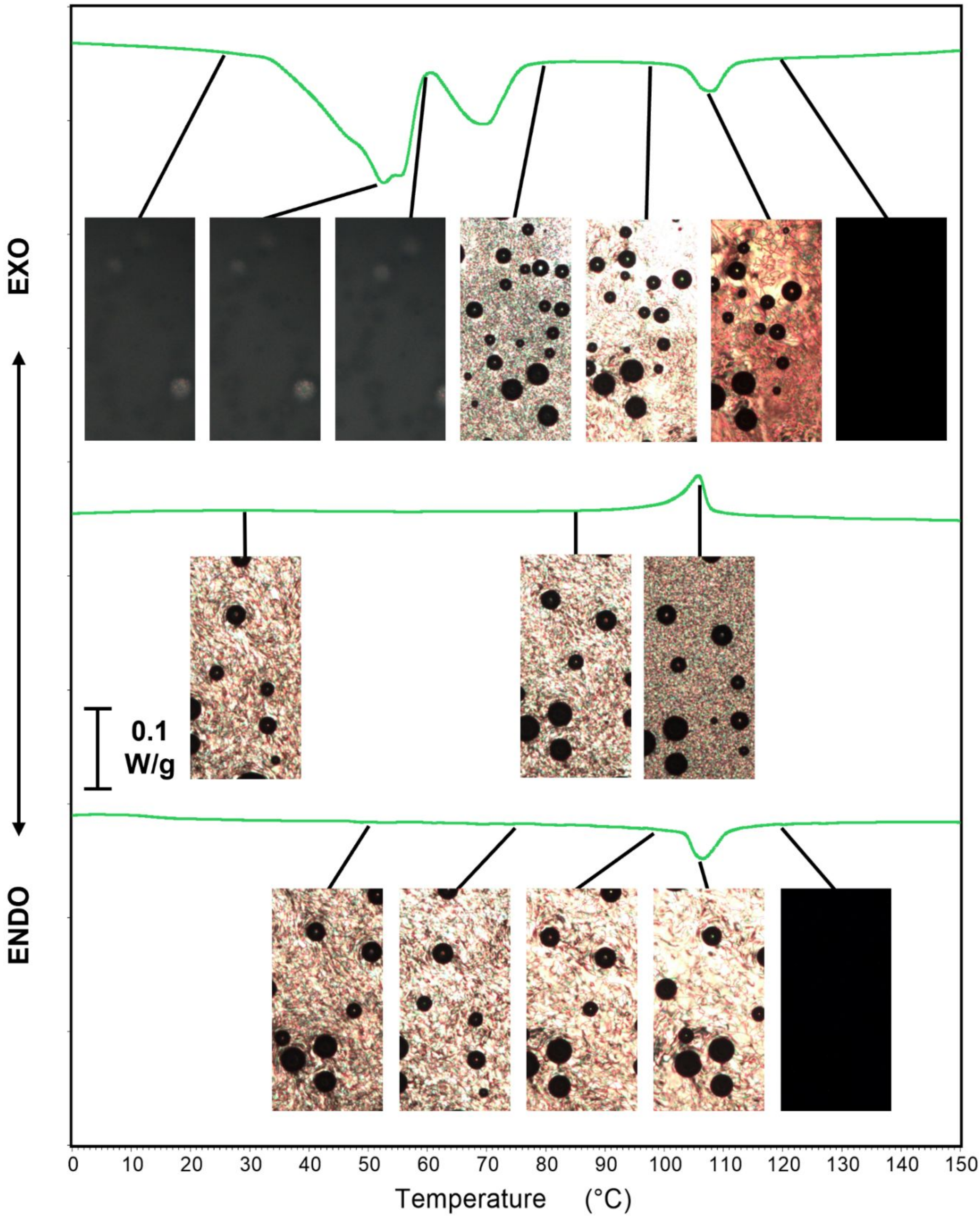

Figure S16. DSC curves and POM photographs of **L2** film, **Table S1** (M_n^{NMR} = 2.26 kDa). From the top, first heating scan (10 °C·min^{-1}, from –90 °C to +200 °C), controlled cooling (10 °C·min^{-1}, from +200 °C to –90 °C), second heating scan (10 °C·min^{-1}, from –90 °C to +200 °C). The temperature at which the POM images were collected was chosen based on the observed transition temperatures. For the first heating scan: T_{CN} = 69 °C, T_{NI} = 106 °C; for the cooling scan: T_{IN} = 106 °C, T_{NC} *undetected*; and for the second heating scan: T_{CN} *undetected*, T_{NI} = 106 °C.

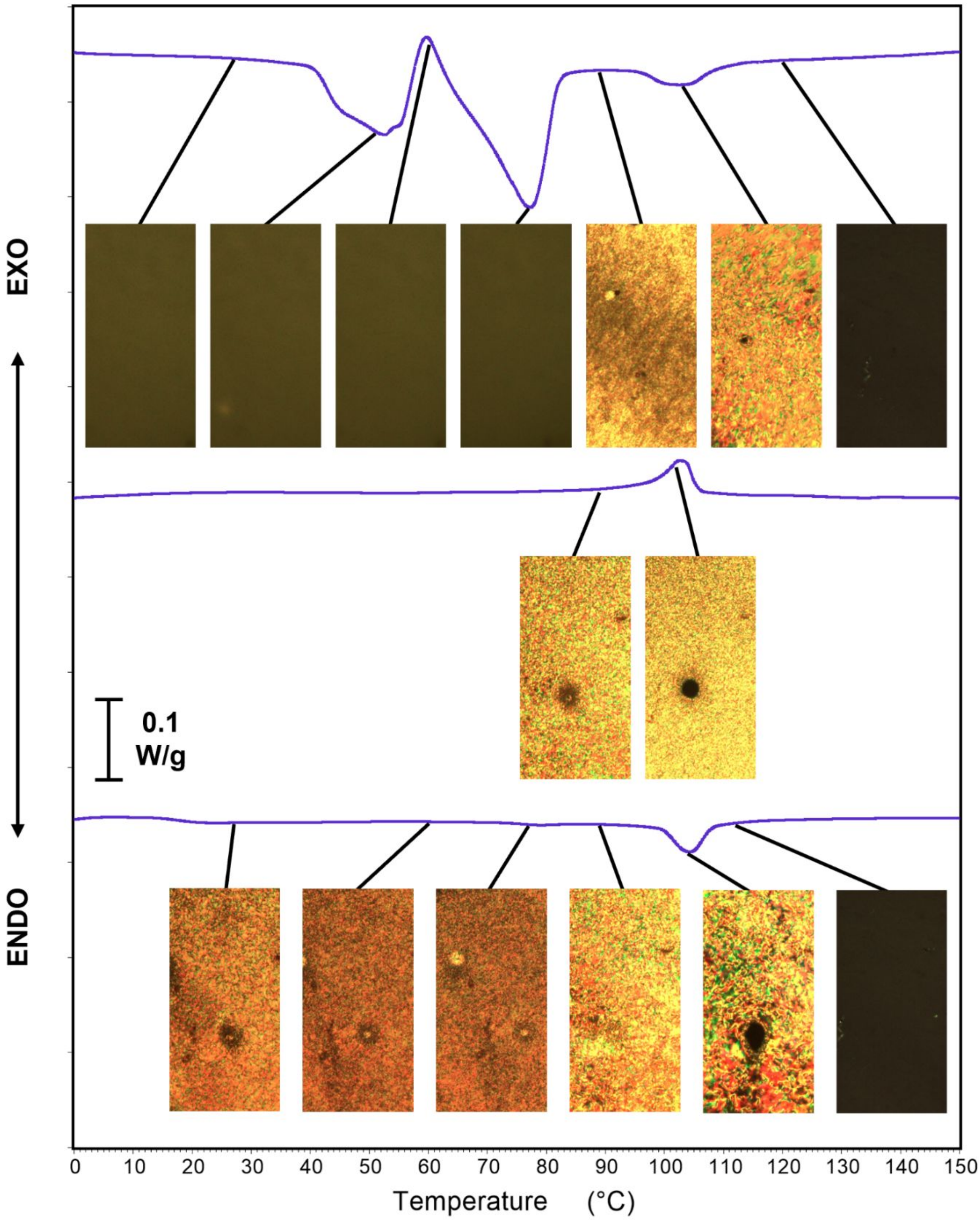

Figure S17. DSC curves and POM photographs of **L3** film, **Table S1** (M_n^{NMR} = 4.08 kDa). From the top, first heating scan (10 °C·min^{-1}, from –90 °C to +200 °C), controlled cooling (10 °C·min^{-1}, from +200 °C to –90 °C), second heating scan (10 °C·min^{-1}, from –90 °C to +200 °C). The temperature at which the POM images were collected was chosen based on the observed transition temperatures. For the first heating scan: T_{CN} = 77 °C, T_{NI} = 104 °C; for the cooling scan: T_{IN} = 103 °C, T_{NC} *undetected*; and for the second heating scan: T_{CN} *undetected*, T_{NI} = 104 °C.

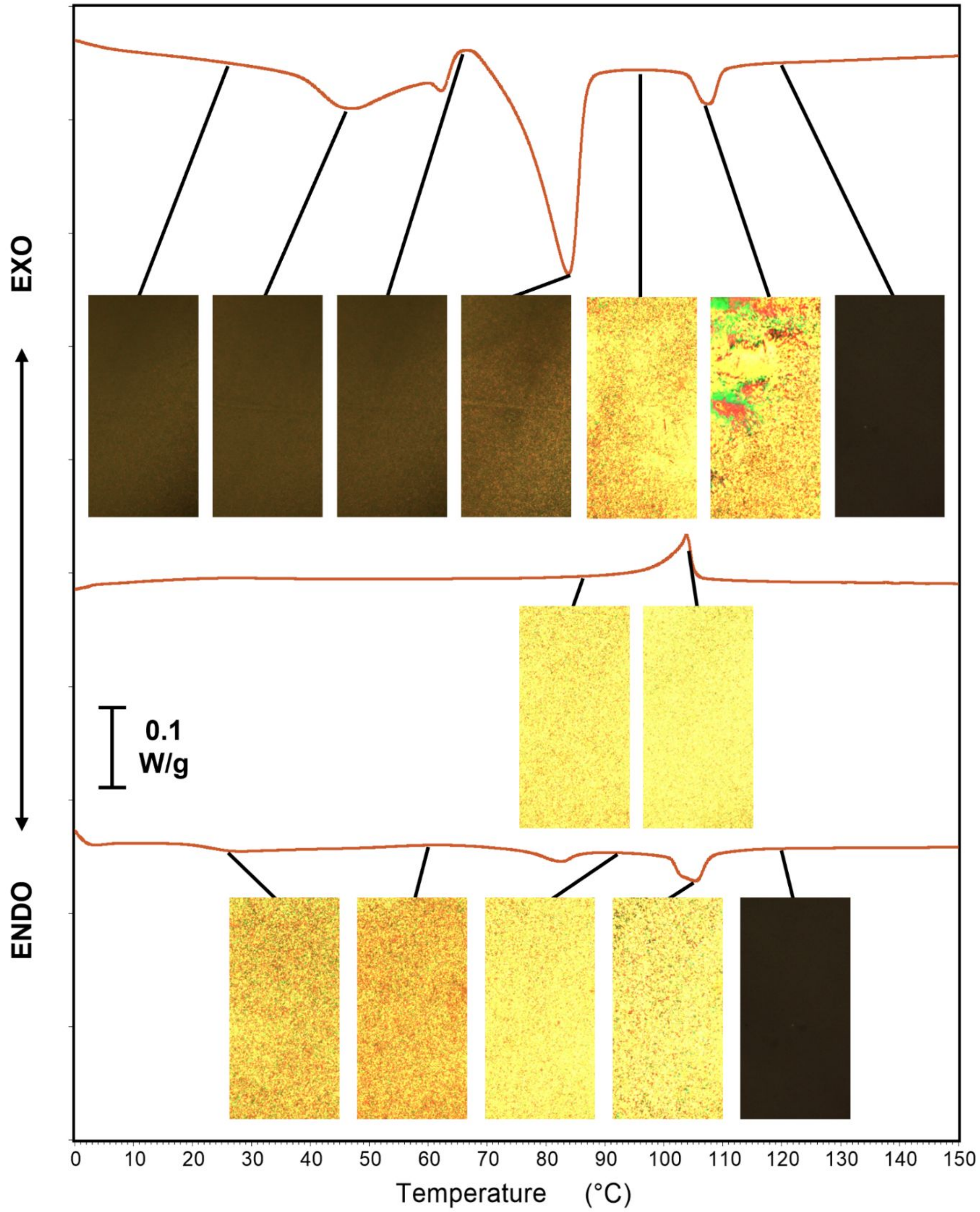

Figure S18. DSC curves and POM photographs of **L4** film, **Table S1** (M_n^{NMR} = 7.68 kDa). From the top, first heating scan (10 °C·min^{-1}, from –90 °C to +200 °C), controlled cooling (10 °C·min^{-1}, from +200 °C to –90 °C), second heating scan (10 °C·min^{-1}, from –90 °C to +200 °C). The temperature at which the POM images were collected was chosen based on the observed transition temperatures. For the first heating scan: T_{CN} = 84 °C, T_{NI} = 105 °C; for the cooling scan: T_{IN} = 104 °C, T_{NC} *undetected*; and for the second heating scan: T_{CN} *undetected*, T_{NI} = 105 °C.

3. Characterization and properties of branched oligomers and residual linear precursors (B and M)

Table S2. Morphology of the electrospun mats obtained on a static collector from solutions of oligomers pre-cured with varying amounts of PeTT (**B**).[a]

entry	Precursor [b] (M_n^{NMR})	$2 \cdot N_{PeTT} \cdot N_L^{-1}$ [c] (%)	Morphology [d]	*Gel Content* [e] (%)
**B1**	**L2** (2.26 kDa)	20	*Beads*	*n.d.*
**B2**		25	*Beaded Fibers*	*n.d.*
**B3**		30	*Beaded Fibers*	*n.d.*
**B4**		40	*Fibers*	68.9 ± 3.9
**B5**	**L3** (4.08 kDa)	15	*Beaded Fibers*	*n.d.*
**B6**		25	*Beaded Fibers*	62.9 ± 3.5
**B7**		30	*Fibers*	58.9 ± 2.7
**B8**		40	*Fibers*	62.8 ± 4.2
**B9**	**L4** (7.68 kDa)	10	*Beaded Fibers*	58.0 ± 2.1
**B10**		20	*Fibers*	57.3 ± 2.0
**B11**		30	*Fibers*	50.3 ± 1.8
**B12**		40	*Fibers*	51.3 ± 4.9

a) Common conditions: DBU = 1.5% w/w respect to the amount of linear precursor (w_L), $V_{DCM} = 5 \cdot w_L$ mL mg^{-1}, T = 40 °C, *time* = 24 h.
b) The precursor label refers to **Table S1**.
c) Percent equivalent of PeTT added to the solution, given the chemical quantity of PeTT (N_{PeTT}) and that of the precursor (N_L).
d) Morphology assessed by optical microscopy. Common ES-PC conditions: concentration of **B** = 0.3 g mL^{-1} in chloroform, TX:DBU=4%:8% w/w with respect to **B**, feeding rate = 1 mL·h^{-1}, collector = static, potential = 20 kV, needle-collector distance = 20 cm, UV lamp-collector distance = 50 cm, IR lamp = OFF, temperature = 26 °C.
e) Calculated with **Equation 6** for fibrous mats, while not determined (*n.d.*) for compositions that resulted in electrospray.

Table S3. Screening of the electrospinning and photocuring conditions by varying the use of an IR lamp, $[\text{oligomer}]_0$, and the amounts of TX and DBU (**M**). [a] Color code: green for mats with well-defined fibrous morphology and high birefringence; blue for mats with good morphology but low birefringence; yellow for mats with film-like morphology and high birefringence; white for mats with film-like morphology and low birefringence.

entry	Oligomer (Precursor, $2{\cdot}N_{PeTT}{\cdot}N_L^{-1}$)	ρ_X^{th} [b] (mmol g^{-1})	TX:DBU [c] (w/w%)	*Fiber Quality*[d]	*Birefringence Quality*[d]	*Gel Content* [e] (%)	T_g [f] (°C)
**M01** [g]		0.373	8 : 8	*low*	*high*	78.6 ± 0.7	27.0
**M02**		0.373	4 : 8	*low*	*low*	74.0 ± 3.0	20.3
**M03** [g]		0.373	4 : 11	*low*	*low*	68.4 ± 1.7	13.9
**M04**		0.373	8 : 12	*high*	*high*	78.4 ± 4.6	28.4
**M05** [h]		0.373	16 : 50	*low*	*low*	*n.d.*	33.9
**M06** [h]		0.373	16 : 20	*high*	*high*	82.8 ± 9.1	41.9
**M07**	**B7**	0.373	8 : 50	*low*	*low*	*n.d.*	29.2
**M08**	(**L3**, 30%)	0.373	8 : 20	*high*	*high*	76.0 ± 4.5	31.1
**M09**		0.373	12 : 35	*low*	*high*	69.5 ± 5.2	35.4
**M10** [h]		0.373	12 : 5	*high*	*low*	87.0 ± 6.6	38.7
**M11** [h]		0.373	12 : 10	*high*	*high*	83.9 ± 5.8	39.0
**M12** [h]		0.373	12 : 15	*high*	*high*	80.1 ± 6.0	39.6
**M13**		0.373	12 : 20	*high*	*high*	74.5 ± 4.9	38.0
**M14**		0.373	12 : 25	*low*	*high*	68.5 ± 4.0	36.1
**M15** [h]		0.594	12 : 10	*low*	*low*	*n.d.*	*n.d.*
**M16** [h]	**B4**	0.594	12 : 15	*low*	*high*	*n.d.*	*n.d.*
**M17**	(**L2**, 40%)	0.594	12 : 20	*low*	*high*	*n.d.*	*n.d.*
**M18** [i]		0.594	12 : 20	*high*	*low*	80.5 ± 4.0	43.8
**M19** [i]		0.594	9.3 : 15.6	*high*	*high*	*n.d.*	39.7
**M20**	**B10**	0.220	12 : 20	*high*	*high*	72.8 ± 1.9	36.0
**M21** [j]	(**L4**, 20%)	0.220	12 : 20	*low*	*high*	68.3 ± 2.1	40.1

a) The electrospun oligomer mixture employed were **B7**, **B4**, and **B10** (**Table S2**). Common ES-PC conditions: concentration of **B** = 0.35 g mL^{-1} in chloroform, total volume = 1 mL, feeding rate = 1 mL h^{-1}, collector = rotating drum (⌀ = 3 cm, spinning at 5000 RPM) wrapped in parchment paper, potential = 20 kV, needle-collector distance = 20 cm, UV lamp-collector distance = 35 cm, IR lamp = ON positioned at 17 cm from the collector (collector surface temperature = 50 °C), chamber temperature = 26 °C.
b) Calculated with **Equation 4**.
c) With respect to the amount of **B**.
d) Each mat was classified according to the outcomes of SEM and POM analysis: the green color was assigned to mats with well-defined fibrous morphology exhibiting strong birefringence, the blue color corresponded to mats with good morphology but low birefringence, the yellow color corresponded to film-like morphology with high birefringence, while mats with film-like morphology and low birefringence remained uncolored.
e) Calculated with **Equation 6**, while not determined (*n.d.*) for compositions that resulted in non-cohesive mats. In other words, mats that, once submerged in DCM, do not hold the shape and crumble.
f) Determined by DSC from the second heating scan (10 °C min^{-1}).
g) IR lamp = OFF.
h) Once homogenized, the **B**:TX:DBU:Chloroform mixture turns opaque due to precipitation of TX.
i) $[\mathbf{B4}]_0$ = 0.45 g mL^{-1}.
j) $[\mathbf{B10}]_0$ = 0.25 g mL^{-1}.

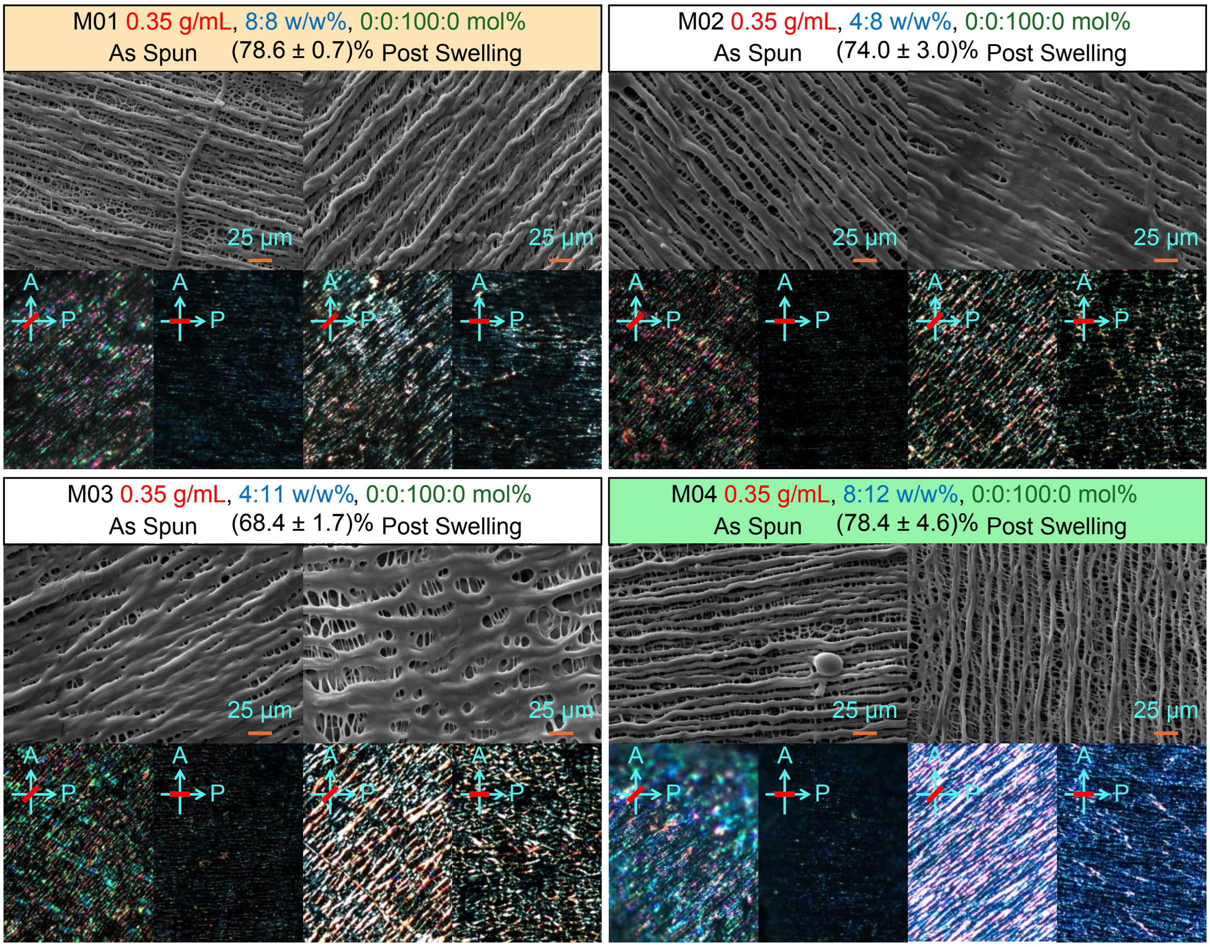

Figure S19. SEM and POM images of electrospun mats **M01**–**M04** (**Table S3**). The variables distinguishing each mat are indicated above the images: oligomer concentration in $CHCl_3$ (*red*), TX:DBU weight ratio relative to the oligomer (*blue*), and molar fraction of linear precursors **L1**:**L2**:**L3**:**L4** (*green*). Furthermore, in **M01** and **M03** the IR lamp was turned off, meanwhile in **M02** and **M04** the IR lamp was turned on. Common ES-PC conditions are reported in **Table S3**. The gel content is also reported (*in parentheses, black*). The SEM images of both as-spun (*left*) and post-swelling mats (*right*) are reported (scale bar = 25 µm). Similarly, the POM images show the mats with fibers rotated 45° relative to the polarization direction and the mat with fibers aligned with the polarization direction, both as-spun and post swelling in DCM. Color code: green for mats with well-defined fibrous morphology and high birefringence; yellow for mats with film-like morphology and high birefringence; white for mats with film-like morphology and low birefringence.

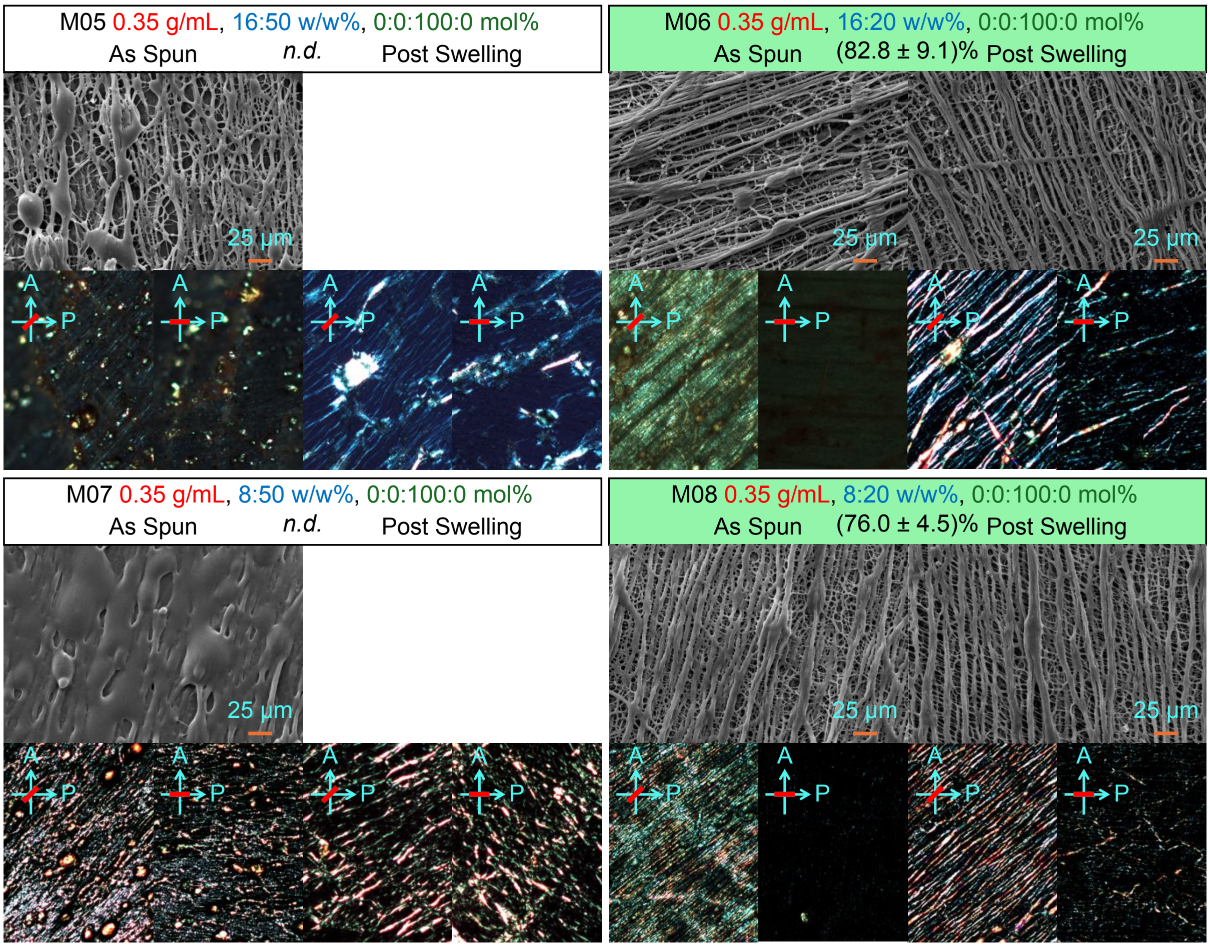

Figure S20. SEM and POM images of electrospun mats **M05**–**M08** (**Table S3**). The variables distinguishing each mat are indicated above the images: oligomer concentration in $CHCl_3$ (*red*), TX:DBU weight ratio relative to the oligomer (*blue*), and molar fraction of linear precursors **L1**:**L2**:**L3**:**L4** (*green*). Common ES-PC conditions are reported in **Table S3**. The gel content is also reported (*in parentheses, black*). The SEM images of both as-spun (*left*) and post-swelling mats (*right*) are reported (scale bar = 25 μm). The POM images show the mats with fibers rotated 45° relative to the polarization direction and the mat with fibers aligned with the polarization direction, both as-spun and post swelling in DCM. Note that we could not collect SEM images for post swelling **M05** and **M07** since these were non-cohesive mats. In other words, mats that, once submerged in DCM, do not hold the shape and crumble. Color code: green for mats with well-defined fibrous morphology and high birefringence; white for mats with film-like morphology and low birefringence.

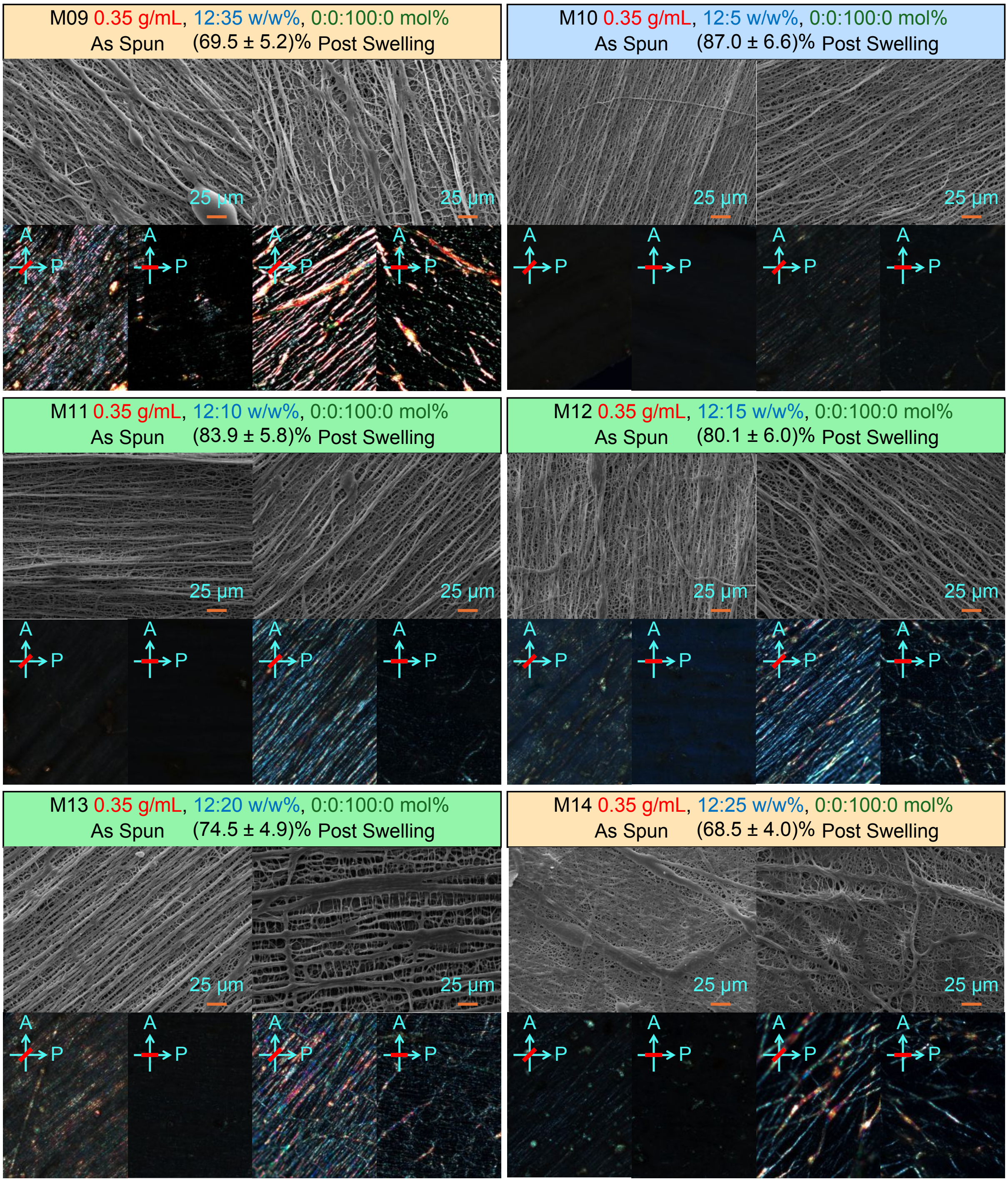

Figure S21. SEM and POM images of electrospun mats **M09**–**M14** (**Table S3**). The variables distinguishing each mat are indicated above the images: oligomer concentration in $CHCl_3$ (*red*), TX:DBU weight ratio relative to the oligomer (*blue*), and molar fraction of linear precursors **L1**:**L2**:**L3**:**L4** (*green*). Common ES-PC conditions are reported in **Table S3**. The gel content is also reported (*in parentheses, black*). The SEM images of both as-spun (*left*) and post-swelling mats (*right*) are reported (scale bar = 25 µm). The POM images show the mats with fibers rotated 45° relative to the polarization direction and the mat with fibers aligned with the polarization direction both as-spun and post swelling in DCM. Color code: green for mats with well-defined fibrous morphology and high birefringence; blue for mats with good morphology but low birefringence; yellow for mats with film-like morphology and high birefringence.

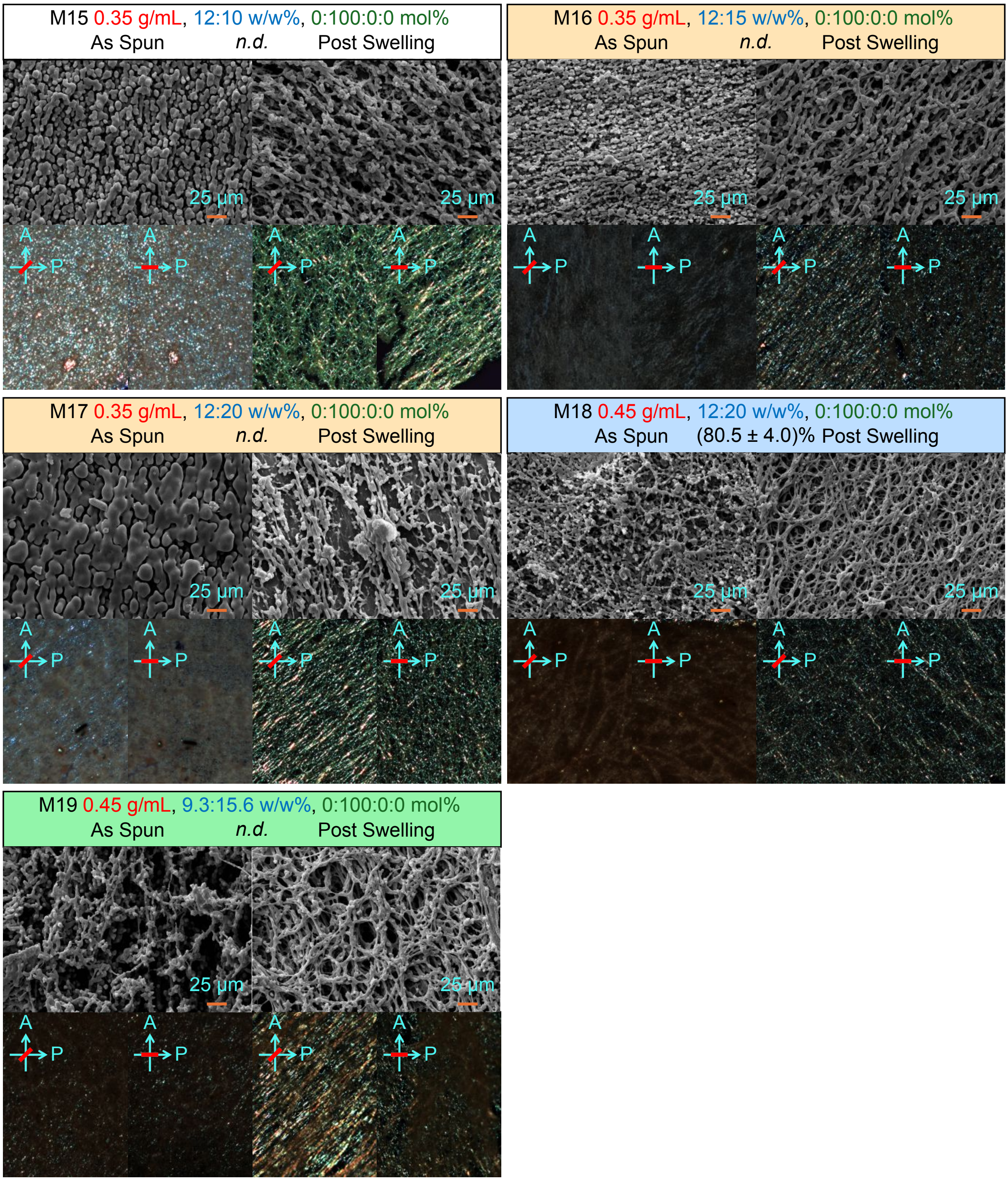

Figure S22. SEM and POM images of electrospun mats **M15**–**M19** (**Table S3**). The variables distinguishing each mat are indicated above the images: oligomer concentration in $CHCl_3$ (*red*), TX:DBU weight ratio relative to the oligomer (*blue*), and molar fraction of linear precursors **L1**:**L2**:**L3**:**L4** (*green*). Common ES-PC conditions are reported in **Table S3**. The gel content is also reported (*in parentheses, black*). The SEM images of both as-spun (*left*) and post-swelling mats (*right*) are reported (scale bar = 25 µm). The POM images show the mats with fibers rotated 45° relative to the polarization direction and the mat with fibers aligned with the polarization direction, as-spun and post swelling in DCM. Color code: green for mats with well-defined fibrous morphology and high birefringence; blue for mats with good morphology but low birefringence; yellow for mats with film-like morphology and high birefringence; white for mats with film-like morphology and low birefringence.

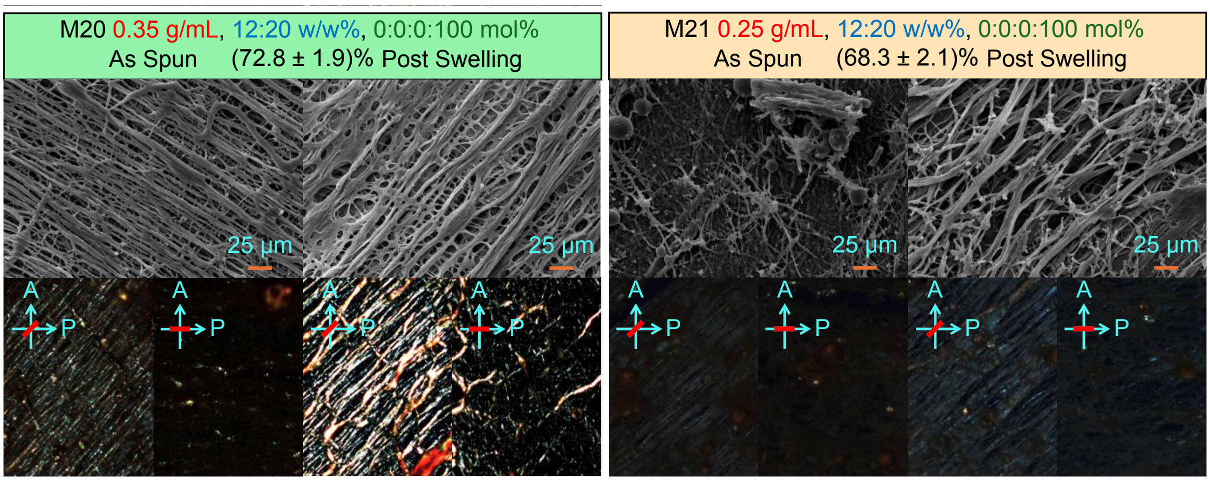

Figure S23. SEM and POM images of electrospun mats **M20**–**M21** (**Table S3**). The variables distinguishing each mat are indicated above the images: oligomer concentration in $CHCl_3$ (*red*), TX:DBU weight ratio relative to the oligomer (*blue*), and molar fraction of linear precursors **L1**:**L2**:**L3**:**L4** (*green*). Common ES-PC conditions are reported in **Table S3**. The gel content is also reported (*in parentheses, black*). The SEM images of both as-spun (*left*) and post-swelling mats (*right*) are reported (scale bar = 25 μm). The POM images show the mats with fibers rotated 45° relative to the polarization direction and the mat with fibers aligned with the polarization direction, as-spun and post swelling in DCM. Color code: green for mats with well-defined fibrous morphology and high birefringence; yellow for mats with film-like morphology and high birefringence.

4. Characterization and properties of mixtures of branched oligomers and residual linear precursors (C)

Table S4. ES-PC of mixtures of oligomers, targeting different theoretical crosslinking densities (ρ_X^{th}).[a]

entry	$\chi_{L1} : \chi_{L2} : \chi_{L3} : \chi_{L4}$	$2 \cdot N_{PeTT} \cdot N_L^{-1}$ (%)	ρ_X^{th} [b] (mmol g^{-1})	[oligomer]$_0$ (g mL^{-1})	TX : DBU [c] (w/w%)	T_g [d] (°C)
**C01**	0.00 : 0.24 : 0.26 : 0.50	27	0.291	0.30	14 : 23.3	41.8
**C02**	0.00 : 0.75 : 0.00 : 0.25	35	0.399	0.37	11.4 : 18.9	36.7
**C03**	0.00 : 0.83 : 0.13 : 0.04	38	0.514	0.45	9.3 : 15.6	34.5
**C04**	0.00 : 0.75 : 0.20 : 0.05	37	0.484	0.42	10 : 16.7	40.4
**C05**	0.00 : 0.54 : 0.30 : 0.16	34	0.397	0.37	11.4 : 18.9	37.2
**C06**	0.00 : 0.20 : 0.75 : 0.05	31	0.386	0.36	11.8 : 19.6	35.7
**C07**	0.81 : 0.16 : 0.02 : 0.01	7.1	1.24	0.45	9.3 : 15.6	32.3
**C08**	0.85 : 0.11 : 0.03 : 0.01	5.4	1.28	0.42	10 : 16.7	26.2
**C09**	0.84 : 0.13 : 0.02 : 0.01	5.9	1.29	0.45	9.3 : 15.6	27.8
**C10**[e]	0.25 : 0.04 : 0.01 : 0.01	2.5	2.23	0.30	14 : 23.3	40.2
**C11**	0.69 : 0.26 : 0.04 : 0.01	12	1.06	0.45	9.3 : 15.6	32.7
**C12**	0.75 : 0.20 : 0.03 : 0.01	9.3	1.15	0.45	9.3 : 15.6	28.5

a) Common ES-PC conditions: total volume = 1 mL (concentration of [oligomer]$_0$ is given in the table), feeding rate = 1 mL h^{-1}, collector = rotating cylinder (⌀ = 3 cm, spinning at 5000 RPM) wrapped in parchment paper, potential = 20 kV, needle-collector distance = 20 cm, UV lamp-collector distance = 35 cm, IR lamp = ON, positioned at 17 cm from the collector (collector surface temperature = 50 °C), chamber temperature = 26 °C.
b) Calculated with **Equation 4**.
c) With respect to the amount of oligomer.
d) Determined by DSC from the second heating scan (10 °C min^{-1}).
e) Neat RM257 was added to the mixture, which accounts for the remaining molar ratio.

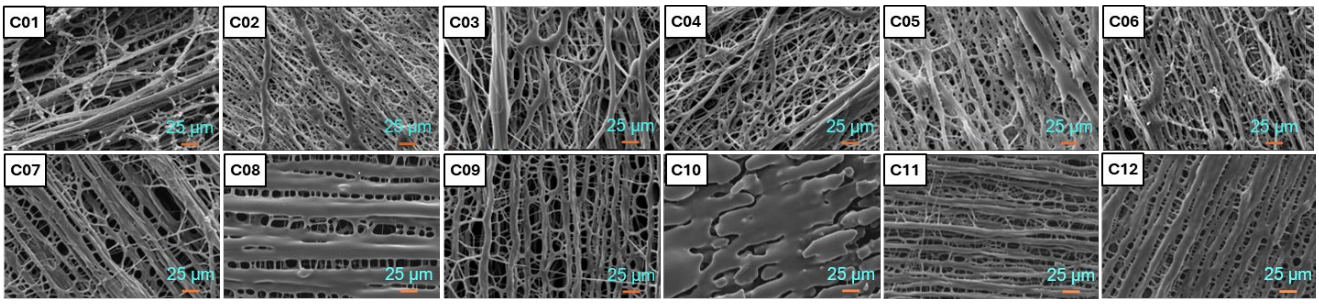

Figure S24. SEM images of electrospun mats **C01–C12** (**Table S4**).

Representative samples from the C series were selected based on their theoretical crosslinking density and mechanically characterized by tensile stress–strain measurements at 125 °C (Figure S25), corresponding to the temperature used for sample pre-stretching during the stress-induced actuation experiments. Among the tested samples, C02, which exhibited the lowest theoretical crosslinking density (ρ_X^{th} = 0.399 mmol g^{-1}), showed the lowest Young's modulus (E = 0.17 MPa). Higher Young's modulus values were measured for C12 (ρ_X^{th} = 1.15 mmol g^{-1}; E = 0.36 MPa) and C09 (ρ_X^{th} = 1.29 mmol g^{-1}; E = 0.39 MPa). As expected, the elastic modulus increased with increasing theoretical crosslinking density, reflecting the greater network connectivity of the more crosslinked samples.

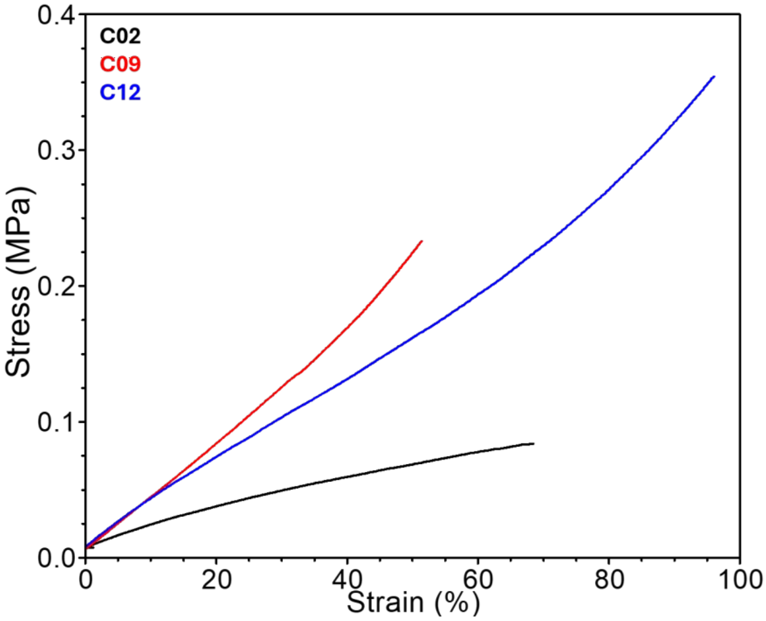

Figure S25. Stress-strain curves of representative samples of C series.

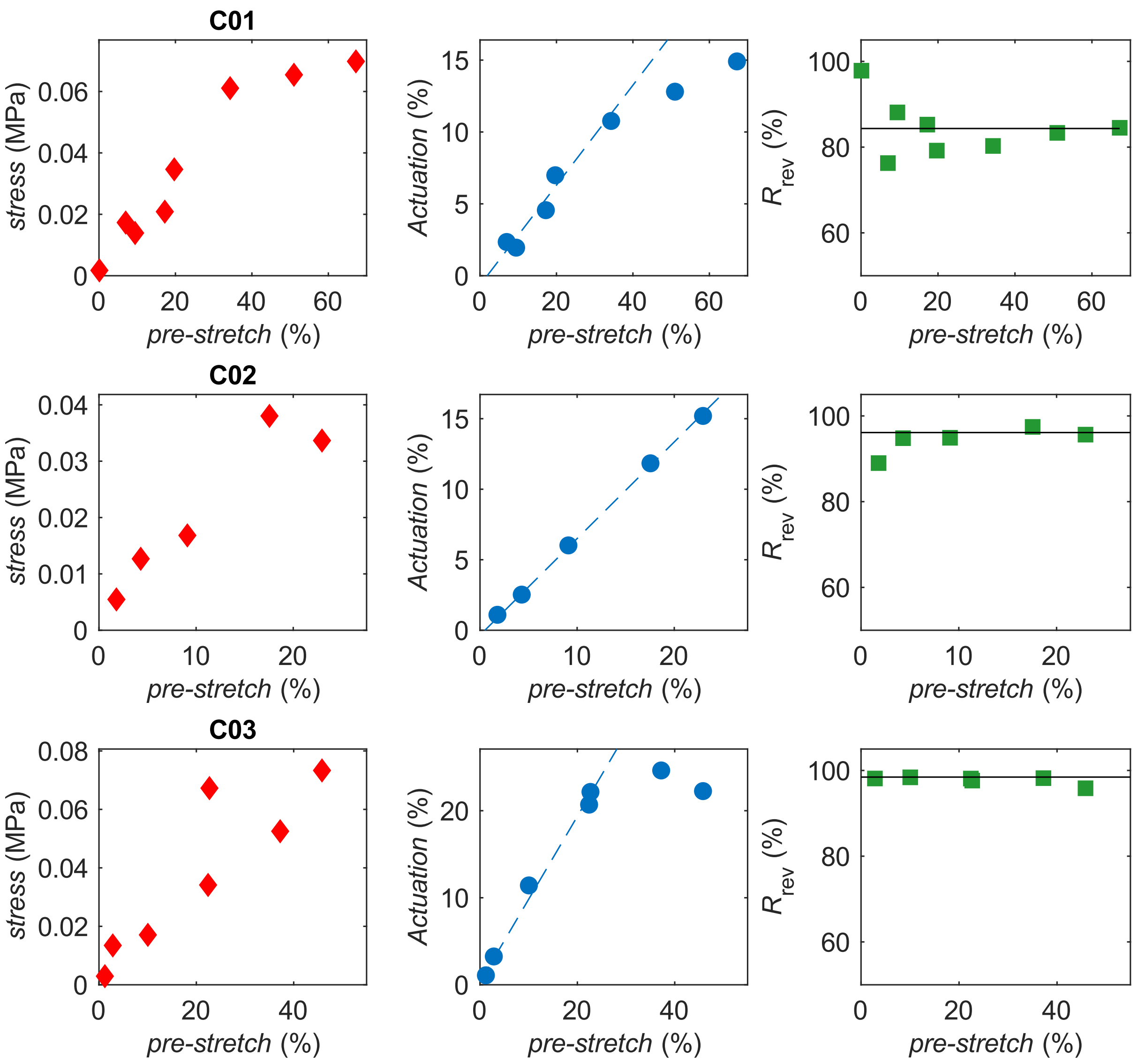

Figure S26. Thermal actuation tests (30–125 °C). The first row: C01 sample; second row C02 sample; third row: C03 sample (**Table S4**). The first column displays the graph of applied stress versus the resulting pre-stretch at 125 °C (*red*), the second column shows the graph of *Actuation* (calculated with **Equation 7**) vs. pre-stretch (*blue*), and the third column presents the graph of R_{rev} (calculated with **Equation 8**) vs. pre-stretch (*green*). The regression lines of *Actuation* were calculated using only the first points in the linear regime, meanwhile the continuous line in R_{rev} represents the mean value.

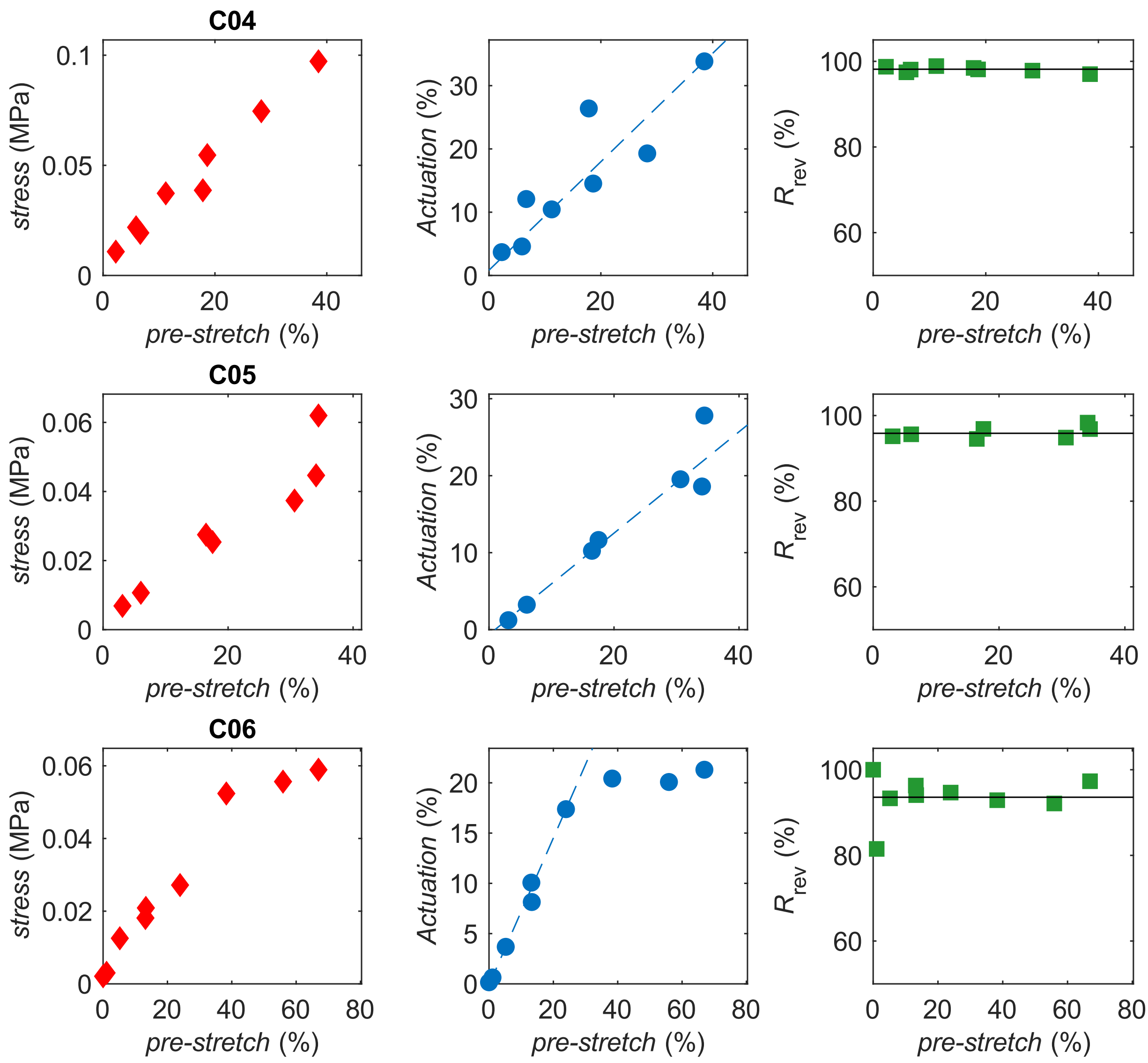

Figure S27. Thermal actuation tests (30–125 °C). The first raw: C04 sample; second raw C05 sample; third raw: C06 sample (**Table S4**). The first column displays the graph of applied stress versus the resulting pre-stretch at 125 °C (*red*), the second column shows the graph of *Actuation* (calculated with **Equation 7**) vs. pre-stretch (*blue*), and the third column presents the graph of R_{rev} (calculated with **Equation 8**) vs. pre-stretch (*green*). The regression lines of *Actuation* were calculated using only the first points in the linear regime, meanwhile the continuous line in R_{rev} represents the mean value.

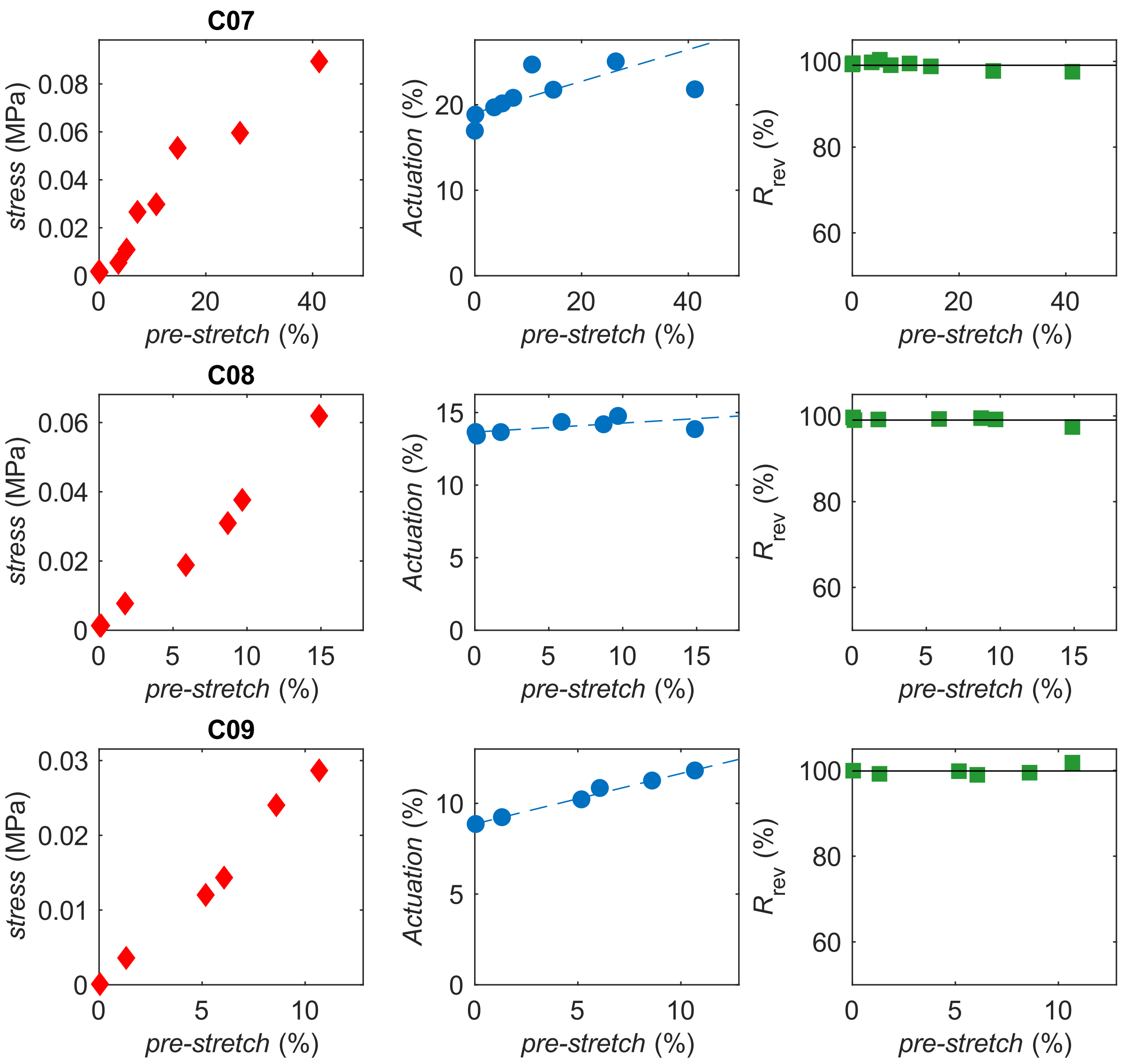

Figure S28. Thermal actuation tests (30–125 °C). The first raw: C07 sample; second raw C08 sample; third raw: C09 sample (**Table S4**). The first column displays the graph of applied stress versus the resulting pre-stretch at 125 °C (*red*), the second column shows the graph of *Actuation* (calculated with **Equation 7**) vs. pre-stretch (*blue*), and the third column presents the graph of R_{rev} (calculated with **Equation 8**) vs. pre-stretch (*green*). The regression lines of *Actuation* were calculated using only the first points in the linear regime, meanwhile the continuous line in R_{rev} represents the mean value.

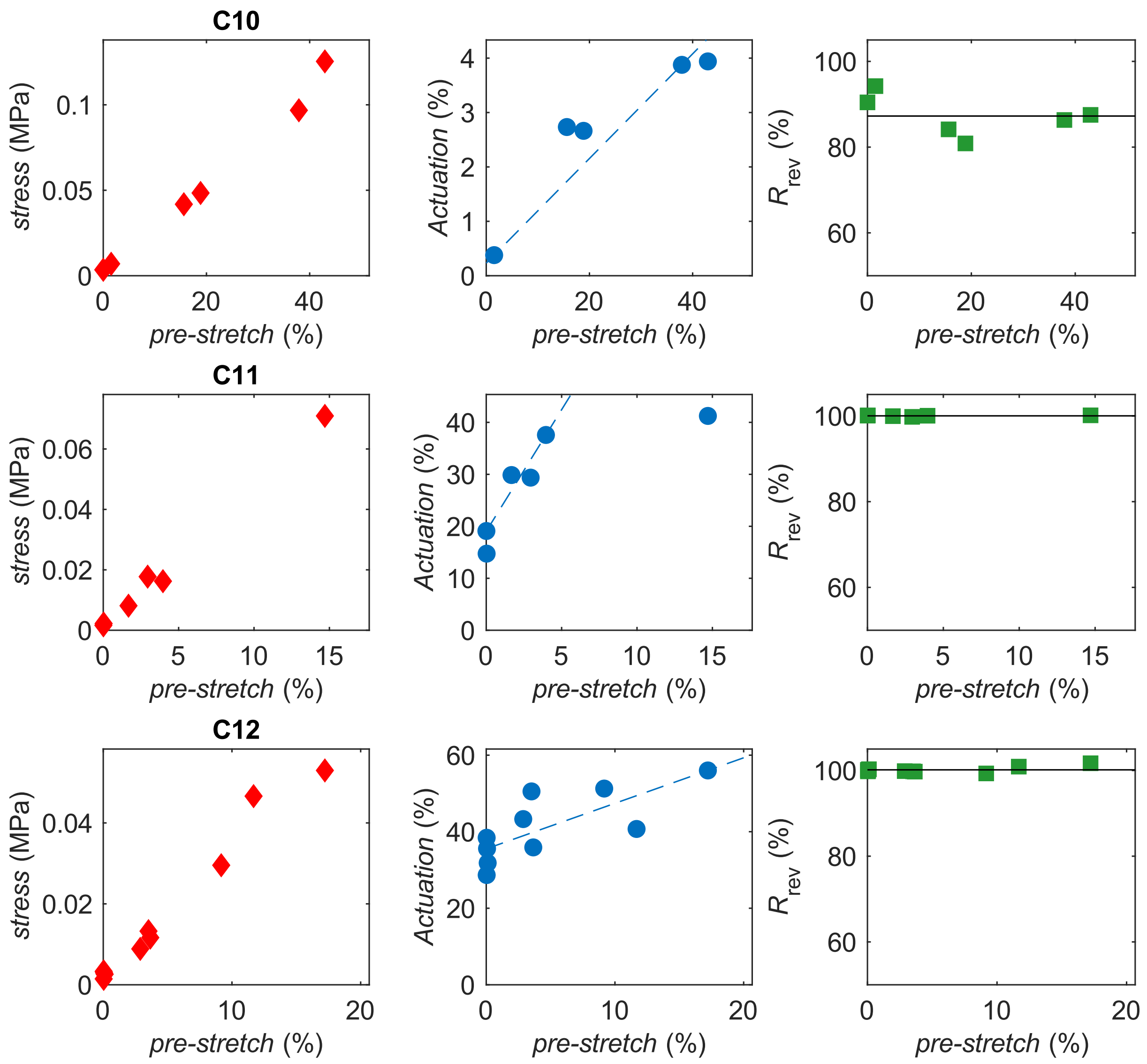

Figure S29. Thermal actuation tests (30–125 °C). The first raw: C10 sample; second raw C11 sample; third raw: C12 sample (**Table S4**). The first column displays the graph of applied stress versus the resulting pre-stretch at 125 °C (*red*), the second column shows the graph of *Actuation* (calculated with **Equation 7**) vs. pre-stretch (*blue*), and the third column presents the graph of R_{rev} (calculated with **Equation 8**) vs. pre-stretch (*green*). The regression lines of *Actuation* were calculated using only the first points in the linear regime, meanwhile the continuous line in R_{rev} represents the mean value.

Table S5. Slope and intercept of the linear correlation between *Actuation* vs. *pre-stretch* for the tested samples together with the corresponding crosslinking density.

Entry	ρ_X^{th} [a] (mmol g^{-1})	Intercept [b]	Slope [b]	*Actuation*^{max} [c] (%)	*Stress-Free Actuation* [d] (%)
C01	0.291	- 0.654	0.348	14.9	- 0.54
C02	0.399	- 0.346	0.685	15.2	- 0.65
C03	0.514	0.310	0.952	24.6	- 0.47
C04	0.484	0.835	0.857	33.8	- 0.9
C05	0.397	- 0.626	0.658	27.8	- 0.71
C06	0.386	- 0.121	0.732	21.3	0.16
C07	1.24	19.03	0.186	25.1	17.9
C08	1.28	13.66	0.062	14.8	13.6
C09	1.29	8.864	0.278	11.8	8.86
C10	2.23	0.231	0.096	3.94	- 0.64
C11	1.06	19.05	4.68	41.2	16.9
C12	1.15	35.52	1.19	56.0	33.6

a) Calculated with **Equation 4**.
b) Determined by linear interpolation of the first datapoints in the Actuation vs. pre-stretch plot (blue data) of **Figure S26-29**.
c) Maximum *Actuation* achieved under stress. Each sample was tested at its maximum pre-stretch before failure.
d) Actuation under stress free condition (pre-stretch = 0%)

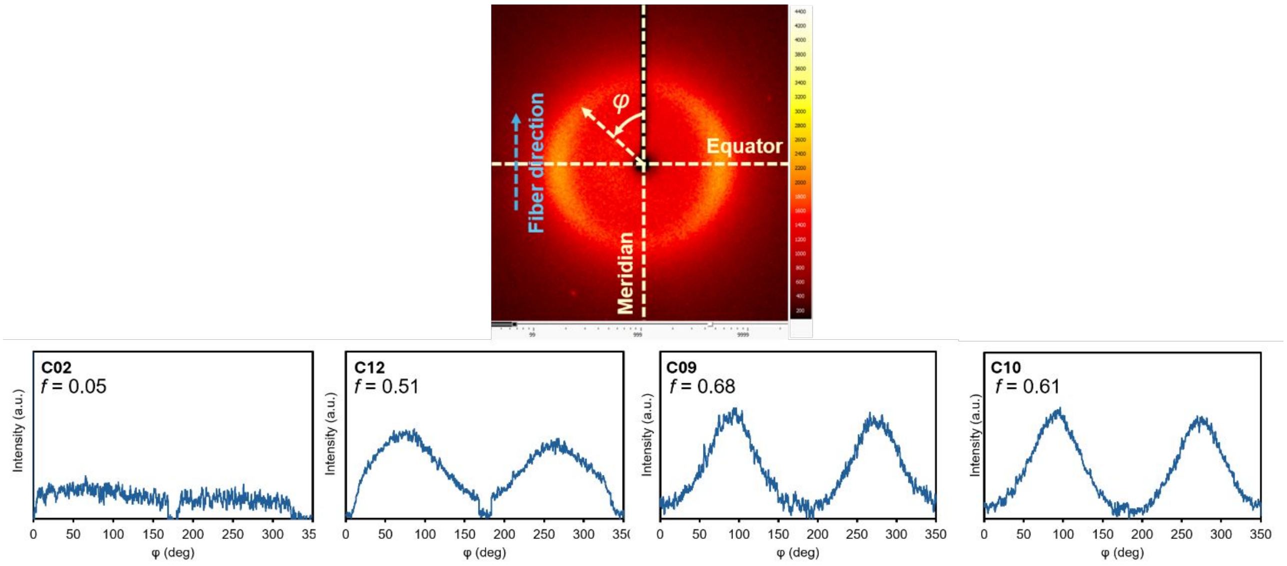

Figure S30. **Top:** representative 2D-WAXD pattern with indications about equator, meridian, and fiber directions. **Bottom:** azimuthal profiles at $2\theta = 19.6°$ of C02, C12, C09, and C10 samples (**Table S5**); the orientation factors, *f*, were determined by applying **Equation 5** and are reported for each tested sample.

DSC analysis before and after photothermal actuation shows a T_g increase of 15 °C in the sample free of HNAB (**Figure S31**). Furthermore, after photothermal actuation, the irradiated spot acquired a darker coloration.

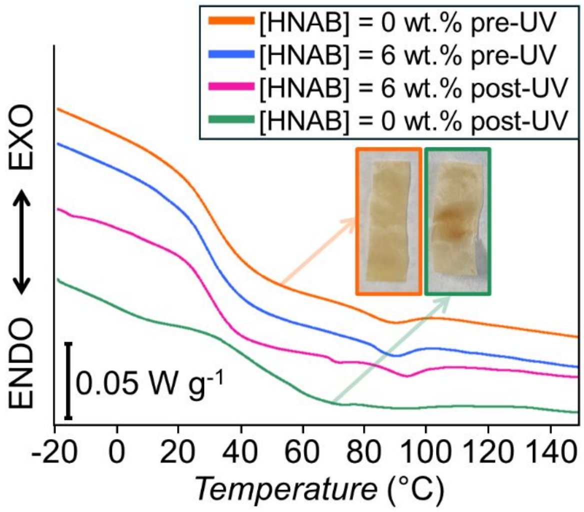

Figure S31. Deleterious effect of UV actuation in the absence of dye, shown by DSC curves and sample photographs before and after photothermal actuation (pristine without and with HNAB, *orange* and *blue*, respectively; UV actuated with and without HNAB, *magenta* and *green*, respectively). The T_g values are [HNAB] = 0 wt.% pre-UV: 31.0 °C, [HNAB] = 6 wt.% pre-UV: 30.5 °C, [HNAB] = 6 wt.% post-UV: 31.0 °C, [HNAB] = 0 wt.% post-UV: 46.0 °C.

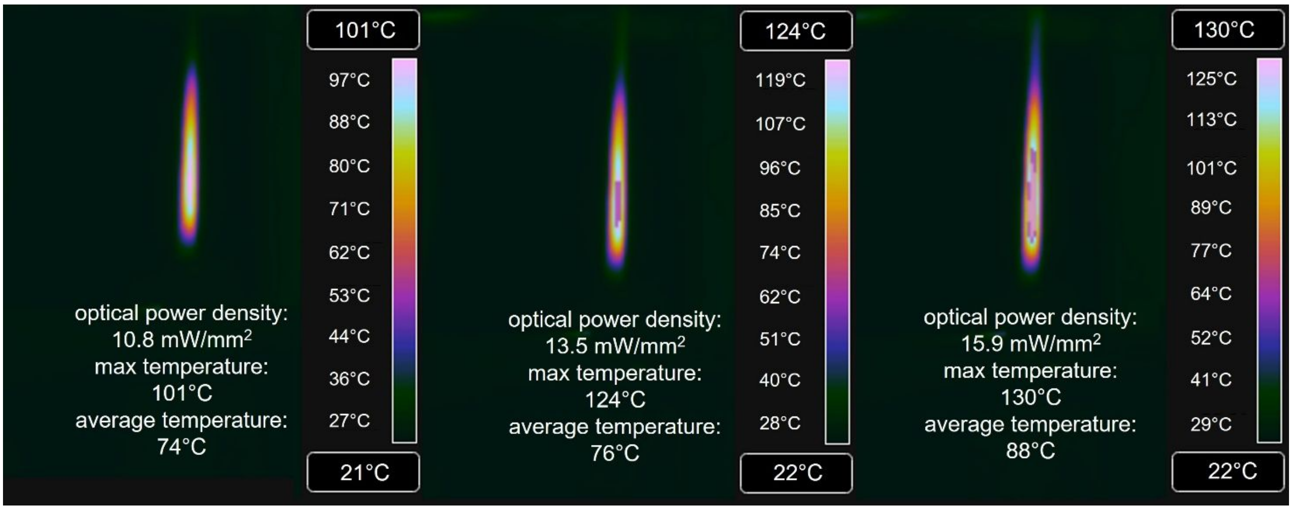

Figure S32. Thermal images of LCEs during photo-actuation test under visible light at three different optical power densities. The test was carried out under the same conditions as the photoactuation setup, using three optical power densities during a stimulation of 5 s.

5. Supporting Video

Sample C12 was placed on a microscope slide coated with silicone oil and mounted on the heating stage (THMS600 with a T90 controller, Linkam) to cycle the temperature from 30 °C to 130 °C. The video was recorded under the polarized optical miscroscope (Zeiss Axioscope) equipped with a Zeiss Axiocam 208 color and analyzed with Zen 3.6 software.

Video S1. Stress-free thermal actuation of sample C12.
